# Predictors for Long-Term Survival After Resection of Pancreatic Ductal Adenocarcinoma: A Systematic Review and Meta-Analysis

**DOI:** 10.1245/s10434-024-15281-1

**Published:** 2024-05-06

**Authors:** Ammar A. Javed, Omar Mahmud, Asad Saulat Fatimi, Alyssar Habib, Mahip Grewal, Jin He, Christopher L. Wolfgang, Marc G. Besselink, for the PANC-PALS Consortium

**Affiliations:** 1 NYU Langone Health, NYU Grossman School of Medicine, New York, USA; 2 Department of Surgery, Amsterdam UMC, Location University of Amsterdam, Amsterdam, The Netherlands; 3 Cancer Center Amsterdam, Amsterdam, The Netherlands; 4 Medical College, Aga Khan University, Karachi, Pakistan; 5 Department of Surgery, Johns Hopkins School of Medicine, Baltimore, USA

**Keywords:** Pancreatic neoplasms, PDAC, Pancreatic ductal carcinoma, Long-term survival, Postoperative survival, Meta-analysis, Systematic review, Pancreatic diseases

## Abstract

**Background:**

Improved systemic therapy has made long term (≥ 5 years) overall survival (LTS) after resection of pancreatic ductal adenocarcinoma (PDAC) increasingly common. However, a systematic review on predictors of LTS following resection of PDAC is lacking.

**Methods:**

The PubMed, Embase, Scopus, and Cochrane CENTRAL databases were systematically searched from inception until March 2023. Studies reporting actual survival data (based on follow-up and not survival analysis estimates) on factors associated with LTS were included. Meta-analyses were conducted by using a random effects model, and study quality was gauged by using the Newcastle-Ottawa Scale (NOS).

**Results:**

Twenty-five studies with 27,091 patients (LTS: 2,132, non-LTS: 24,959) who underwent surgical resection for PDAC were meta-analyzed. The median proportion of LTS patients was 18.32% (IQR 12.97–21.18%) based on 20 studies. Predictors for LTS included sex, body mass index (BMI), preoperative levels of CA19-9, CEA, and albumin, neutrophil-lymphocyte ratio, tumor grade, AJCC stage, lymphovascular and perineural invasion, pathologic T-stage, nodal disease, metastatic disease, margin status, adjuvant therapy, vascular resection, operative time, operative blood loss, and perioperative blood transfusion. Most articles received a “good” NOS assessment, indicating an acceptable risk of bias.

**Conclusions:**

Our meta-analysis pools all true follow up data in the literature to quantify associations between prognostic factors and LTS after resection of PDAC. While there appears to be evidence of a complex interplay between risk, tumor biology, patient characteristics, and management related factors, no single parameter can predict LTS after the resection of PDAC.

**Supplementary Information:**

The online version contains supplementary material available at 10.1245/s10434-024-15281-1.

Pancreatic ductal adenocarcinoma (PDAC) remains one of the leading causes of all cancer-related deaths globally.^1^ Approximately 20% of patients have resectable disease at diagnosis, with an additional 30% having borderline or locally advanced PDAC who can potentially undergo resection after neoadjuvant or induction therapy.^2,3^ A majority of patients undergoing successful resection experience early disease recurrence which is predominantly systemic.^4^ This suggests a need for effective systemic control if we are to improve long-term survival.^5,6^

Although there is extensive literature analyzing the outcomes of patients with PDAC after surgical resection, the rarity of long-term survival (≥ 5 years; LTS) has resulted in a lack of data on this subgroup of patients and most studies report estimates of LTS using survival analysis methods.^7^ Moreover, due to the low rates of LTS and the challenge of completing long-term follow-up after surgery, studies that do assess true LTS are typically limited by their small sample sizes. This has hindered the accurate quantification of associations between clinicopathological factors and biomarkers that may predict long-term survival in these patients.

Reliable prognostication is a prerequisite for patients to be able to understand the ramifications of their diagnosis and engage in meaningful dialogue with their physicians.^8^ This is especially true in the context of PDAC, where surgery is associated with considerable morbidity and mortality.^9^ In addition, enhancing the ability to accurately predict long-term survival is critical for measuring the impact of newly researched therapies on patient outcomes.^8^

With the development and increased utilization of effective systemic therapies, an increase in the rate of LTS is expected. There is thus an evident need to identify factors predictive of true 5-year survival in patients with PDAC following resection in the current era of therapy. The aim of this systematic review and meta-analysis was to pool the available data in the literature on actual 5-year survival after resection of PDAC to accurately characterize predictors of true LTS.

## Methods

### Study Design

This review was conducted in accordance with the Preferred Reporting Items for Systematic Reviews and Meta-Analyses (PRISMA) statement and was pre-registered with the National Institute for Health Research PROSPERO International prospective register of systematic reviews with the registration ID “CRD42022346060.” A completed PRISMA checklist (2020) is presented in Supplementary Section 1.

### Search Strategy

#### Eligibility Criteria

Included were studies that compared patients who achieved LTS after resection of PDAC with those who did not. Excluded were studies in languages other than English, and conference abstracts.

#### Screening and Data Extraction

All studies identified by the search strategy were deduplicated and independently screened for relevance based on the abstract by two reviewers (O.M. and A.S.F.), who were blinded to each other’s decisions. Discrepancies between the reviews were resolved via consensus or, when needed, by a third reviewer (A.A.J.). The remaining studies were then screened on a full-text basis by two reviewers (O.M. and A.S.F). Again, discrepancies were resolved by a third reviewer (A.A.J.). The remaining eligible studies were selected for extraction of usable data. Relevant data were extracted to a spreadsheet by both reviewers and discrepancies were corrected (O.M. and A.S.F.). Extracted data included general article information and clinicopathological data of long-term survivors and non-long-term survivors.

Projected estimates of 5-year survival (i.e., LTS), such as from Kaplan-Meier/survival analyses, were excluded. All analyzed data was based on patients followed after resection of PDAC, and all LTS patients were those who achieved actual survival of least 5 years. Some studies had adequately long durations such that patients reported as “at-risk” at 60 months of follow-up, as part of Kaplan-Meier analysis, did represent true LTS patients. However, to take this value to capture all LTS patients in such studies would be to assume that any patients who underwent right-censoring, such as due to loss to follow-up or late enrolment, would not have survived for 5 years. This assumption would have led to underestimated LTS rates in these studies. More importantly, in the analysis of factors associated with LTS, this approach could have skewed the measures of effect towards or away from the null depending on which group had more such patients. Consequently, such data were not incorporated or pooled in the analysis.

#### Approximating Means and Standard Deviations for Nonparametrically Distributed Data

From the included studies, nonparametrically distributed continuous data were available as either sample medians and interquartile ranges or sample medians and ranges. Strategies outlined by Wan et al.^10^ were used to approximate means and standard deviations from medians, ranges, and interquartile ranges to enable them to be pooled as continuous data for meta-analyses. These methods are more generalizable for cases where the sample size *n* is small compared with other methods to estimate means and standard deviations from medians, interquartile ranges, and ranges that are independent of *n*. The continuous data that were approximated using these methods have been indicated as such in Supplementary Section 3.

#### Continuity Corrections for Zero-Event-Studies

In addition to requests for data made to corresponding authors, further efforts were made to include as much data as possible for the meta-analyses. Where studies reported 0 events for dichotomous variables, continuity corrections were used to include their data in the meta-analysis equations. For studies that reported 0 events in either arm, otherwise called “single-zero-event studies,” continuity corrections of 0.5 were automatically added to the arms by Review Manager version 5.4.1 (The Cochrane Collaboration, Copenhagen, Denmark). For studies that reported 0 events in both arms for dichotomous variables, otherwise called “double-zero-event studies,” Carter estimators, as outlined by Wei et al.^11^ were utilized; continuity corrections of 1 were added to the number of events in both arms, and 2 was added to the total number of participants in each of both arms. This approach was necessary as excluding double zero events may introduce estimation biases to the overall effect sizes and has been shown to impact conclusions since they are not necessarily noninformative.^12,13^

#### Quality Assessment (Risk of Bias) and Certainty of Evidence Assessment

The risk of bias in individual studies was assessed using the Newcastle Ottawa scale (NOS) for observational studies. Two reviewers (A.H. and M.G.) independently assessed each included study using these tools and scored them accordingly. The scores were then compared, and discrepancies resolved by consulting a third reviewer (O.M.) or by consensus amongst the authors. The certainty of evidence for each outcome was determined by using the GRADE (Grading of Recommendations, Assessment, Development and Evaluation) approach and recorded independently by two reviewers (A.H. and M.G.) via the GRADE Pro Software (McMaster University and Evidence Prime Inc, Ontario, Canada).^14^ In cases of disagreement, a third reviewer was consulted (A.S.F.). Publication bias was assessed by generating funnel plots for outcomes reported in more than ten studies.

### Statistical Analysis

All meta-analyses were performed using the Review Manager v5.4.1 software (The Cochrane Collaboration, Copenhagen, Denmark). A minimum of two studies were required to perform a given meta-analysis. Analyses of continuous variables were synthesized as mean differences (MD) with 95% confidence intervals (CI) while pooled odds ratios (OR) with 95% CI were used for categorical variables. Forest plots were generated for all meta-analyses with two or more included studies. For outcomes where the results obtained depended on the definition of an event, the alternate approaches to pooling the data have all been taken and the results reported (tumor grade, AJCC stage, adjuvant therapy). For outcomes where statistical heterogeneity was attributed to important differences between the meta-analyzed studies, subgroup analyses were performed and reported (neoadjuvant therapy, major vascular resection). The characteristics used to define these subgroups are reported in a case-by-case manner in the results section. Finally, for any outcome where sensitivity analysis found that the statistical significance of the overall result depended on data from a particular study, the methodology of this study was reviewed to identify potential explanations. However, no such explanations were found, and the studies were not removed from the meta-analyses.

A random effects model was used for all meta-analyses in anticipation of potential heterogeneity between studies. Nontrivial heterogeneity was found in some outcomes. This might be accounted for, in part, by differences in the characteristics of included participants, operative procedures, and the exact chemotherapeutic agents used in adjuvant and neoadjuvant therapies. Heterogeneity was assessed by using the τ^2^ and I^2^ statistics.

All reported *p*-values are two-tailed, and a value less than 0.05 has been considered statistically significant.

## Results

### Literature Search

**Fig. 1:**
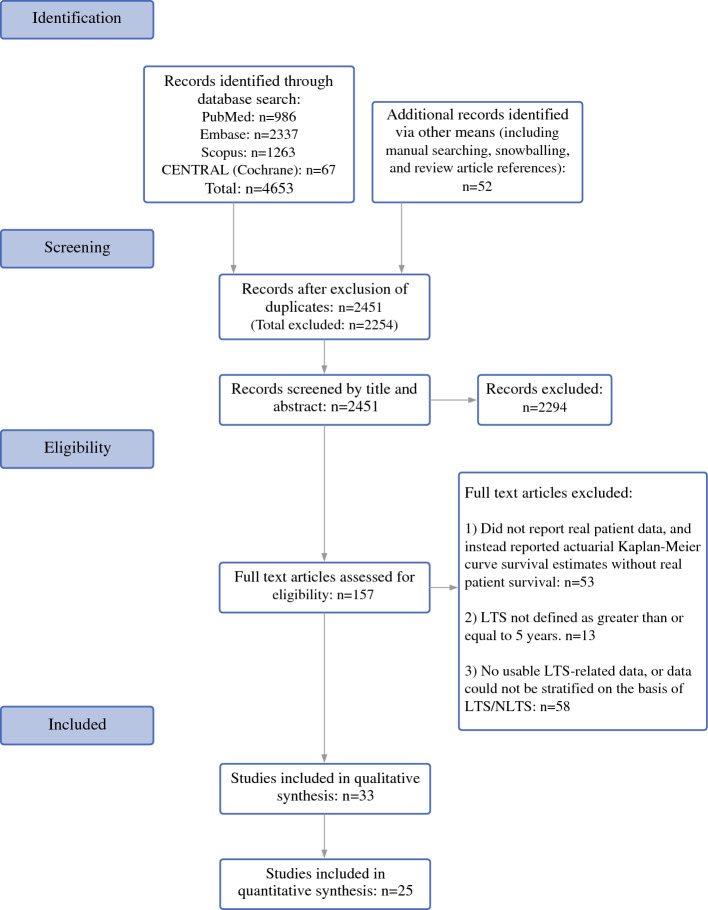
PRISMA flowchart

**Fig. 2:**
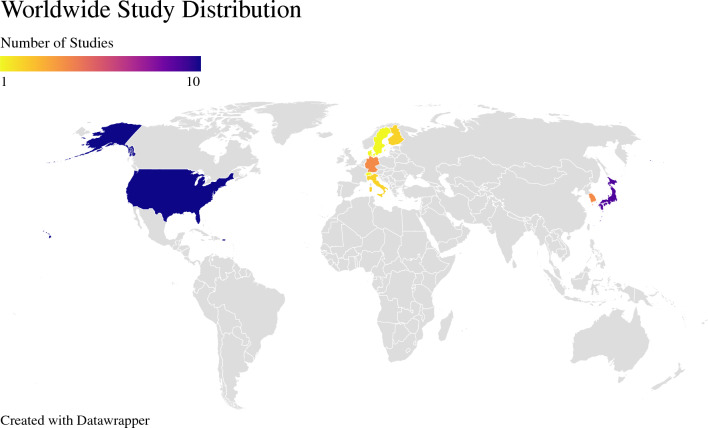
Worldwide distribution of studies

Overall, 33 studies fulfilled the eligibility criteria.^7,15–46^ Of these, 25 could be quantitatively meta-analyzed with a total of 27,091 patients (LTS: 2,132 [7.87%], non-LTS: 24,959 [92.13%]).^15–29,31–40^ A diagrammatic representation of our search and article selection strategy is represented in Fig. 1. Of the 33 studies included, 9 were from North America, 10 from Europe, 13 from Asia, and 1 from both North America and Europe. The regional distribution of included studies is depicted in Fig. 2. All included studies were from countries classified as high-income by the World Bank as of 2023. Study and participant characteristics are presented in Tables 1 and 2.

**Table 1:**
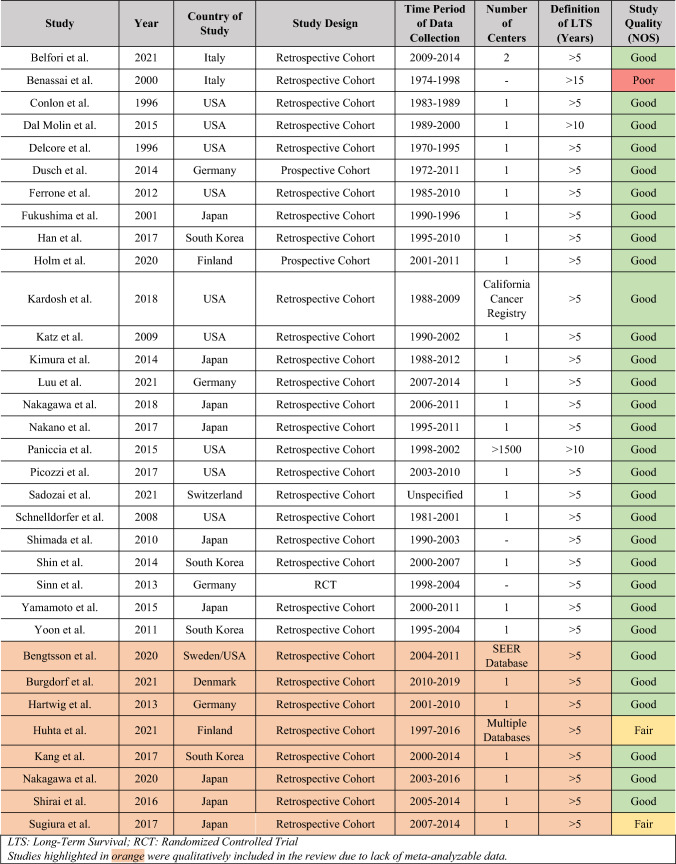
Characteristics of included studies

**Table 2:**
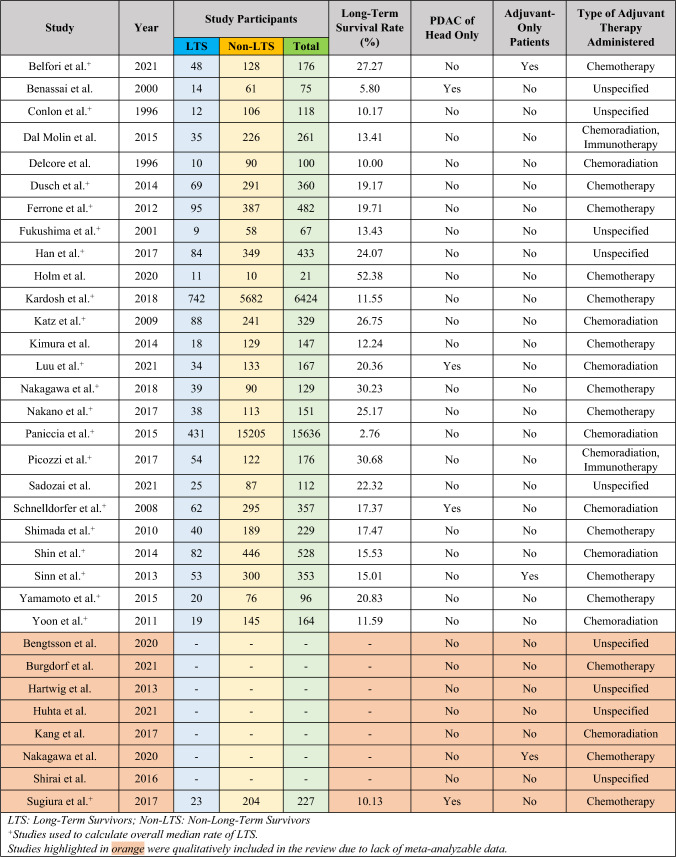
Participant characteristics

### Risk of Bias Assessment

Based on the NOS, the overall risk of bias in the majority of studies (30/33) was assessed to be low with a resultant “good” study quality. Of the remaining three studies, two were of “fair” quality due to lower ratings in the comparability and outcome subdomains of the tool, whereas one was of “poor” quality due to subpar reporting of data and methodology. The detailed study quality assessments, including subdomain scores, are available in Supplementary Section 4.

### Proportions of LTS Across Studies

The proportion of patients who achieved LTS was computed for each study where the method of enrollment would not distort this value (such as in cases where equal numbers of LTS and non-LTS patients were retrospectively enrolled and compared). 20 studies (of the 33 reviewed), with a total of 27,091 patients, were deemed to meet this criterion. For each of these 20 studies, the proportion of LTS was calculated and the median was found to be 18.32% (IQR 12.97–21.18%). However, it is not appropriate to consider this an analysis of the rate of LTS, nor was a meta-analyzed rate of LTS computable, because the degree of loss to follow-up and eligibility criteria for participants and management strategy varied significantly across studies. Consequently, the percentage of patients in each study who survived beyond 5 years LTS was affected by factors such as loss to follow-up and exclusion criteria based on postoperative mortality, nodal positivity, margin positivity, metastatic disease, and regimens of chemotherapy utilized. These differences are highlighted in Tables 1 and 2.

### Factors Associated with LTS

The associations between more than 40 clinicopathological factors and LTS are reported in Tables 3 and 4. Associations quantified by our meta-analyses have been classified as patient-, tumor-, and treatment-related variables. Additional evidence and data that were not meta-analyzable have been qualitatively reviewed for all associations in the extended results in Supplementary Section 5, along with additional results. Forest plots for all meta-analyses are reported in the Supplementary Section 6.

**Table 3:** Factors associated with long-term survival after resection of PDAC (continuous variables)

Variable		Total participants	Studies	MD [95% CI] for LTS	*p*	I^2^ (%)	τ^2^	GRADE certainty of evidence
*Continuous variables*								
Clinicopathological factors	Age (years)	19080	11	−1.31 [−3.18, 0.56]	0.17	86	7.24	Very low
	BMI (kg/m^2^)	466	**3**	**1.27 [0.61, 1.92]**	**< 0.001**	28	0.11	Low
Blood chemistry	Preoperative CA19-9 (units/mL)	1034	**6**	**−2601.71 [−3476.62, −1726.80]**	**< 0.001**	99	1042082.35	Very low*
	Preoperative CEA (ng/mL)	794	**4**	**−0.79 [−1.41, −0.17]**	**0.01**	34	0.14	Low
	Preoperative albumin (mg/mL)	695	**3**	**0.22 [0.03, 0.41]**	**0.03**	68	0.02	Very low
	Preoperative bilirubin (mg/dL)	531	2	−1.69 [−3.90, 0.51]	0.13	91	2.33	Very low
	Neutrophil-lymphocyte ratio	171	**1**	**−0.78 [−1.36, −0.19]**	**0.01**	–	–	–
Histopathological factors	Tumor size (cm)	1566	6	−1.45 [−3.19, 0.30]	0.10	99	4.71	Very low
Surgical and management-related factors	Operative blood loss (mL)	964	**4**	**−545.95 [−804.52, −287.39]**	**< 0.001**	71	48192.18	Low
	Operative time (min)	799	**4**	**−33.88 [−59.60, −8.16]**	**0.01**	34	253.27	Moderate
	Hospital stay (days)	660	3	−0.78 [−3.17, 1.61]	0.52	61	2.67	Very low

Statistically significant results (*p* < 0.05) are given in bold

*MD* mean difference; *CI* confidence interval; *LTS* long-term survivors; *BMI* body mass index; *CA19-9* cancer antigen 19-9; *CEA* carcinoembryonic antigen

*Because of very large differences between subgroups, certainty of evidence has been assessed for subgroups separately in the supplement

**Table 4:** Factors associated with long-term survival after resection of PDAC (categorical variables)

Variable		Total participants	Studies	OR [95% CI] for LTS	*p*	I^2^ (%)	τ^2^	GRADE certainty of evidence
*Categorical variables*								
Clinicopathological factors	Female sex (vs. male sex)	21213	**20**	**1.29 [1.01, 1.64]**	**0.04**	72	0.16	Very low
	Biliary stenting	691	3	0.62 [0.24, 1.61]	0.33	76	0.53	Very low
	Diabetes	527	2	0.68 [0.39, 1.18]	0.17	15	0.02	Low
	Preoperative ASA classification (≥3 vs. <3)	655	3	0.80 [0.42, 1.52]	0.5	44	0.14	Low
	Alcohol Use	322	2	0.39 [0.04, 3.61]	0.4	51	1.42	Very low
	Cardiovascular Disease	527	2	1.22 [0.74, 2.02]	0.44	20	0.03	Low
	Hypertension	360	1	0.59 [0.34, 1.03]	–	–	–	–
Histopathological factors and tumor characteristics	Lymph node metastases	13210	**17**	**0.40 [0.35, 0.46]**	**< 0.001**	0%	0.00	High
	Tumour Grade (≥Grade III vs. <Grade III)	19791	**17**	**0.57 [0.43, 0.74]**	**< 0.001**	63	0.14	Low
	AJCC/UICC stage (≥Stage IIB vs. <Stage IIB)	13283	**14**	**0.36 [0.31, 0.41]**	**< 0.001**	0	0.00	High
	AJCC/UICC stage (≥Stage III vs. <Stage III)	13388	**14**	**0.29 [0.20, 0.41]**	**< 0.001**	0	0.00	High
	Pathologic T-stage (≥T3 vs. <T3)	13783	**12**	**0.40 [0.30, 0.53]**	**< 0.001**	37	0.07	Moderate
	Tumour location (pancreatic head vs. other locations)	19631	12	0.96 [0.84, 1.09]	0.5	0	0.00	Low
	Vascular invasion	2526	**10**	**0.52 [0.41, 0.66]**	**< 0.001**	6	0.01	Moderate
	Perineural invasion	2567	**10**	**0.46 [0.29, 0.72]**	**< 0.001**	72	0.37	Low
	Tumour grade (≥Grade II vs. Grade I)	18247	**9**	**0.40 [0.30, 0.54]**	**< 0.001**	46	0.06	Moderate
	Lymphatic invasion	1570	**6**	**0.44 [0.32, 0.60]**	**< 0.001**	13	0.02	Moderate
	Pathologic M-stage	11629	**4**	**0.16 [0.06, 0.38]**	**< 0.001**	0	0.00	High
	CDK2NA mutation	112	1	0.33 [0.07, 1.55]	0.16	–	–	–
	KRAS mutation	112	1	4.60 [0.57, 36.86]	0.15	–	–	–
	SMAD4 mutation	112	1	0.54 [0.11, 2.67]	0.45	–	–	–
	TP53 mutation	112	1	1.18 [0.46, 3.04]	0.74	–	–	–
	Positive resection margins	14212	**19**	**0.42 [0.35, 0.50]**	**< 0.001**	0	0.00	High
Surgical and management-related factors	Adjuvant therapy (chemotherapy and/or radiotherapy)	13574	**13**	**1.75 [1.29, 2.38]**	**< 0.001**	48	0.12	Low
	Adjuvant therapy (chemotherapy only)	1857	10	**1.68 [1.03, 2.74]**	**0.04**	50	0.24	Low
	Vascular resection	1648	**7**	**0.62 [0.41,0.93]**	**0.02**	34	0.10	Low
	Adjuvant Therapy (Any adjuvant therapy including gemcitabine or nab-paclitaxel)	1028	6	2.28 [0.97, 5.35]	0.06	5	0.53	Very low
	Neoadjuvant therapy	795	4	1.48 [0.56, 3.95]	0.43	77	0.67	Very low
	Perioperative blood transfusion	648	**3**	**0.50 [0.33, 0.77]**	**< 0.001**	0	0.00	Moderate
	Major postoperative morbidity/complications	471	3	0.81 [0.47, 1.39]	0.44	0	0.00	Low
	Intraoperative radiotherapy	416	2	1.20 [0.65, 2.22]	0.55	0	0.00	Low

Statistically significant results (*p* < 0.05) are given in bold

*OR* odds ratio; *CI* confidence interval; *LTS* long-term survivors; *AJCC* American Joint Committee on Cancer; *UICC* Union for International Cancer Control

## Patient Characteristics

Pooled data from 11 studies showed no significant difference in the age of patients (in years) who achieved long-term survival after resection and those who did not (MD: −1.31 [−3.18, 0.56]). Additionally, 20 studies were meta-analyzed revealing a significant association between female sex and LTS (OR: 1.29 [1.01, 1.64]).

Three studies reported poolable data on the association between BMI (in kg/m^2^) and LTS. Patients with LTS had a higher mean BMI in all three studies and meta-analysis showed a statistically significant difference between the groups (MD: 1.27 [0.61, 1.92]).

### Serum Biomarkers

#### Preoperative CA19-9

Six studies reporting data on preoperative CA19-9 levels and LTS were meta-analyzed, demonstrating an association between low CA19-9 and LTS (MD: −2601.71 [−3476.62, −1726.80]). The average preoperative CA19-9 levels in LTS patients from these 6 studies ranged from 45 to 159 units/mL, whereas those of patients without LTS ranged from 88 to 380 units/mL. All 6 studies found lower preoperative CA19-9 levels in patients with LTS. However, upon conversion to mean and standard deviation datapoints, extreme heterogeneity was seen in the data when pooled. This occurred due to the extremely large ranges and interquartile ranges associated with the datapoints. The overall mean difference obtained by meta-analyzing these values remained statistically significant and reflects the association between LTS and preoperative CA19-9 levels, but the value and associated confidence interval were not meaningful as a result.

#### Preoperative Carcinoembryonic Antigen

Four studies were meta-analyzed to assess the differences in preoperative carcinoembryonic antigen (CEA) (in ng/mL) between LTS and non-LTS patients, and a significant overall MD of −0.79 [−1.41, −0.17] was obtained.

#### Preoperative Albumin

Three studies reported poolable data comparing pre-operative albumin levels in LTS patients and those without LTS. A significant MD of 0.22 mg/mL [0.03, 0.41] was obtained.

### Histopathological Factors

#### Pathologic T-Stage and Tumor Size

Data from 12 studies were meta-analyzed to compute the association between pathologic T-stage and LTS. With events defined as a pathologic T-stage of 3 or above, a significant negative association with LTS (OR: 0.40 [0.30, 0.53]) was obtained. Six studies provided poolable data on tumor size (in cm). The point estimates of tumor size were lower in LTS than non-LTSs in all pooled studies, but a statistically insignificant overall MD of −1.45 [−3.19, 0.30] was obtained.

#### Tumor Location

Data were pooled from 12 studies to analyze the prognostic value of tumor location, comparing neoplasms occurring in the head of the pancreas with those occurring at other sites. An overall OR of 0.96 [0.84,1.09] was computed, suggesting that tumor location is not a predictor of LTS based on the evidence from these articles. Two of the studies included in this outcome defined LTS as 10 years or greater, while two other studies had disproportionately large sample sizes. However, sensitivity analyses excluding these studies did not significantly alter the calculated result.

#### Nodal Metastases

Data from 17 studies were pooled to assess the odds of finding N1 versus N0 status in LTS patients. An OR of 0.40 [0.35, 0.46] was obtained.

#### Lymphatic, Vascular, and Perineural Invasion

Meta-analysis of data from six studies showed that the odds of finding lymphatic invasion were markedly lower in LTS patients and a statistically significant OR of 0.44 [0.32, 0.60] was obtained. Vascular invasion was found to have a negative relationship with LTS; data from ten studies was pooled to obtain an OR of 0.52 [0.41, 0.66]. Furthermore, ten studies reported poolable data on the odds of finding perineural invasion in LTS patients versus non-LTS patients. A statistically significant overall OR of 0.46 [0.29, 0.72] was computed.

#### Resection Margins

Data from 19 studies were meta-analyzed to calculate the association between R1 or R2 versus R0 margins and LTS. Significantly lower odds of positive resection margins were noted in the LTS group (OR: 0.42 [0.35, 0.50]).

#### Tumor Grade

Tumor grade data were dichotomized and meta-analyzed in two alternate ways to compute LTS ORs. The results of both approaches are reported. Data from nine studies were pooled in the first approach. Moderately, poorly, and undifferentiated tumors were compared as a group with well-differentiated tumors. An overall OR of 0.40 [0.30, 0.54] was obtained.

Data from 17 studies were meta-analyzed in the second approach. Poorly differentiated and anaplastic tumors were considered one category and well or moderately differentiated tumors another, yielding an overall OR of 0.57 [0.43, 0.74].

#### AJCC/UICC Stage

The data on tumor staging was meta-analyzed using two different approaches. Thirteen studies were poolable in the first approach, where events were defined as tumors staged as IIB or higher with an overall OR of 0.36 [0.31, 0.42]. A slightly different set of 13 studies was meta-analyzable in the second approach, where the cutoff was set at stage III leading to an overall OR of 0.29 [0.20,0.41].

#### Distant Metastases

Pooling data from four studies, distant metastases were significantly associated with poorer LTS (OR: 0.16 [0.06, 0.38]).

#### Tumor Mutational Status

The effects of four mutations, namely KRAS, TP53, SMAD4, and CDK2NA were reviewed but data were not poolable for meta-analysis. Sadozai et al. reported data that were used to calculate ORs for KRAS, TP53, CDK2NA, and SMAD4 mutations, which were found to be 4.60 [0.57, 36.86], 1.18 [0.46, 3.04], 0.33 [0.07, 1.55], and 0.54 [0.11, 2.67] respectively.^34^ Thus, no conclusions can be drawn regarding the utility of detecting these mutations to help predict LTS in resected PDAC.

## Management-Related Factors

### Neoadjuvant/Induction Therapy

Four studies reported meta-analyzable data on the association between the use of neoadjuvant therapy and LTS, with an OR of 1.48 [0.56, 3.95]. However, significant statistical heterogeneity was noted in the result (I^2^ = 77%, $\tau$^2^ = 0.67) and important methodological differences between these included studies were identified. Consequently, subgroup analyses were performed.

Three studies investigating patients who received neoadjuvant therapy included both resectable and borderline resectable patients. All three individually, along with the meta-analyzed result, showed no significant association between neoadjuvant therapy and LTS in these cohorts. Nakano et al. only administered neoadjuvant therapy to patients with T3/T4 tumors and reported a significant positive association between neoadjuvant therapy and LTS (OR: 5.12 [2.04, 12.08]).^31^

### Major Vascular Resection

Data from seven studies, of which six reported insignificant findings, were meta-analyzed as a composite outcome that defined the resection of major blood vessels as an event. A significant association with LTS was seen, with an OR of 0.62 [0.41, 0.93], showing that patients who survived >5 years had lower odds of having undergone major vascular resection. Two studies were sub-grouped under portal venous resection only, with an OR of 0.79 [0.36, 1.72]. Two studies were subgrouped as portal venous and/or superior mesenteric venous resection, with an OR of 0.51 [0.11, 2.38]. The three remaining studies were subgrouped with events defined as any vascular resection, and an OR of 0.62 [0.41, 0.93] was computed.

### Operative Time, Blood Loss, and Perioperative Blood Transfusion

Meta-analysis of four studies reporting operative time showed an MD of −33.88 min [−59.60, −8.16], with shorter surgeries being significantly associated with LTS. Data from four studies were meta-analyzed and LTS patients were found to have significantly less operative blood loss (in mL) than non-LTS patients (MD: –545.95 [−804.2, −287.39]). Only one of three pooled studies independently showed a significant association between decreased odds of blood transfusion perioperatively in patients who survived long-term versus those who did not. However, the point estimates for all three studies supported such an association, and meta-analysis showed a significant result overall (OR: 0.50 [0.33, 0.77]).

### Adjuvant Therapy

Some studies reporting the association between adjuvant therapy and LTS did not distinguish between patients receiving one of or both chemotherapy and radiation (Table 1). Furthermore, several studies were conducted before the introduction of more contemporary chemotherapeutic agents, such as gemcitabine and nab-paclitaxel. As such, three alternate meta-analyses were performed on data regarding the effect of adjuvant therapy.

In the first approach, data were pooled from 13 studies and events were defined as the administration of adjuvant chemotherapy and/or radiation to patients. An overall OR of 1.75 [1.29, 2.38] was seen, suggesting an association between this management approach and increased chances of LTS.

In the second approach, nine studies where patients were specifically stated to have received chemotherapy alone were analyzed. A significant result was obtained, with an OR of 1.68 [1.03, 2.74].

Finally, six studies where adjuvant therapy that included gemcitabine or nab-paclitaxel were pooled and an insignificant OR of 2.28 [0.97, 5.35] was obtained. However, this approach was not comprehensive as many studies did not provide specific details on the components of their chemotherapy regimens or did not specify the subgroups of patients who received gemcitabine or nab-paclitaxel. As such, this result should be interpreted with caution and the trend towards an effect size noted (test for overall effect: *p* = 0.06).

## Discussion

Albeit historically dismal, reported rates of LTS after resection for PDAC have consistently increased.^7^ A steady decline in perioperative mortality has enabled studies to be conducted with extended follow-up to identify long-term survivors and the factors associated with this outcome.^27^ This systematic review and meta-analysis found a median proportion of LTS of 18.32% after resection of PDAC. Meta-analysis identified several predictors of LTS: patient related factors (female sex and BMI), serum biomarkers (preoperative CA19-9, CEA, neutrophil-lymphocyte ratio, and albumin levels), histopathological factors (pathologic T-stage, nodal and distal metastases respectively, lymphovascular and perineural invasion, margin status, tumor grade, and AJCC stage), and management-related factors (vascular resection, operative time, operative blood loss, perioperative blood transfusion, and adjuvant therapy).

Although the prognosis of patients diagnosed with PDAC remains poor, there is growing evidence that the rate of LTS is increasing. Large database studies have identified increased rates of LTS after surgical resection, with more recent estimates of 12%, 17%, and 18% in the best of circumstances.^7,25,32^ These figures closely agree with the median rate of LTS that we identified in the literature (18%). However, these studies span multiple decades, lack data after 2011, and report information from registries using ICD codes, which may be unreliable. Among studies that reconfirmed the histopathologic diagnosis of included patients, variable rates of LTS ranging from 16% to 27% were reported.^20,26,37^ This reflects the significant variation seen in the literature across study designs, time periods, and patient populations. Moreover, these studies tend to have smaller sample sizes, limiting precision and generalizability. Thus, our review establishes the need for global collaboration between high-volume centers to provide current data on the trends in LTS in the context of patients’ disease and management.

Our meta-analysis identified associations between the clinicopathologic characteristics of long-term versus non-long-term survivors. Among patient-level factors, biological sex may play a role in determining LTS in PDAC. Previous research investigating the survival difference between sexes has suggested a possible role of antiproliferative effects of estrogen signaling mediated by G-protein coupled receptors, as opposed to nuclear steroid receptors.^47^ This may represent a potential therapeutic target, although further research is needed to establish the importance of this mechanism. However, our results suggest that patient factors are less influential than tumor related factors in determining LTS.

Several serum biomarkers were found to be predictors of LTS. Although the size of the overall mean difference in CA19-9 levels we computed is not meaningful, given the challenges experienced in pooling the data described previously, the association with LTS is well-supported by evidence in the literature from previous studies and survival estimates. Preoperative CA19-9 levels correlate with the presence of extra-pancreatic disease and tumor resectability, though the latter effect is not fully reflected in our results as only patients able to undergo surgery were included.^48–54^ However, much of the prognostic utility of CA19-9 arises from comparing levels seen preoperatively, postoperatively, and during follow-up. Failure of CA19-9 levels to decline sufficiently is indicative of metastases and the need for appropriate further management, while resurgence of levels during follow-up can be a marker of recurrence.^48,55^ The paucity of data quantifying the successive changes in CA19-9 levels in patients achieving LTS precluded our ability meta-analyze their association, and such data should be emphasized as a desired product of future studies. Hyperbilirubinemia and diabetes are also clinically useful markers as they can reflect mass effects of and pathological paracrine signaling between PDAC and normal tissue.^56^ However, our results do not indicate that they are predictive of LTS.

Regarding tumor-related factors, an important result obtained is that the presence of metastatic disease massively hinders patients’ chances of extended survival (OR: 0.16 [0.06, 0.38]) but does not completely eliminate the possibility of LTS. This validates continued emphasis on optimized systemic therapy in appropriate patient populations. Interestingly, it was observed in our results that while increasing pathologic T-stage was significantly associated with decreased odds of LTS (OR: 0.40 [0.30, 0.53]), an insignificant result was obtained for the pooled MD in tumor size between LTS and non-LTS patients (MD: −1.45 cm [−3.19, 0.30]). However, it is pertinent to note that the point estimates of all six studies included in the meta-analysis of tumor size supported an association between smaller tumors and increased survival. It is considered that, because of the marked heterogeneity among these studies, an insignificant overall result might have been obtained in the presence of a true association, thus explaining the apparent discrepancy between these related results.

Data from two included studies showed no significant associations were identified between LTS and various genetic mutations, including P53, KRAS, CDK2NA, and SMAD4. These are among the most commonly mutated/inactivated genes in PDAC, with KRAS mutations present in more than 90% of cases.^56^ Differences between LTS and non-LTS patients’ immune responses to PDAC, such as the proportions of tumor-associated fibroblasts and composition of leukocyte infiltrates in the tumor microenvironment, are currently under investigation.^57^ Such variations may help explain the role of and differences in the effectiveness of the host response to PDAC. However, they also have been shown to affect the cellular uptake of chemotherapy and its eventual efficacy in these patients.^58^ Similar effects have also been associated with derangements in tumor cell metabolism. For example, low cellular ceramide to sphingosine-1-phosphate ratios have also been associated with poorer survival due to increased resistance to agents such as gemcitabine.^59^ Such findings may eventually help to explain treatment failure in particular patients, identify novel therapeutic targets, and clarify the causal determinants of long-term survival in PDAC at a finer level.

Early diagnosis and treatment, including surgical resection and optimized systemic therapy, are critical for bolstering patients’ odds of achieving LTS. Our results show that achieving negative surgical margins and the successful administration of adjuvant chemotherapy are predictive of achieving LTS. Furthermore, our results showed that patients with LTS were more likely to undergo surgery where major vessel resection is not required and where operative time, blood loss, and the need for perioperative blood transfusion were minimal. These associations may reflect superior prognoses in patients with less extensive tumor involvement. Ultimately, it is worth noting that the improvements seen over time in PDAC survival are due in large part to the major advances in controlling operative morbidity and mortality, where high-volume centers now often report mortality rates below 1%.^27,60^ In this context, our results support the notion that local disease control through successful surgery paired with effective systemic therapy are key to prolonged patient survival in PDAC.

Neoadjuvant therapy has become increasingly important in the management of PDAC. While our pooled analysis overall showed no significant association between the use of neoadjuvant therapy and LTS, the interpretation of the subgroups within this analysis is far more meaningful. This review only included resected patients and does not capture the benefit of neoadjuvant therapy in enabling some patients, who may otherwise not be surgical candidates, to undergo resection. Neoadjuvant therapy is hypothesized to primarily benefit patients by eliminating micro-metastases disseminated from the primary tumor.^61,62^ However, evidence does not support the use of neoadjuvant therapy in all patient populations. Harm may occur in cases where the adverse effects of NAT or disease progression worsen patient health or, in some cases, close the window of opportunity to perform curative intent resection.^61^ Currently, the literature seems to point toward improved survival in patients with borderline resectable or locally advanced disease treated with neoadjuvant or induction therapy but not in those considered resectable at baseline.^63^ In line with this, the study by Nakano et al. included in our meta-analysis only administered neoadjuvant therapy to patients with T3/T4 tumors and a significant improvement in LTS was obtained (OR: 5.12 [2.04, 12.86]).^61,64^ In summary, safe surgical intervention combined with effective and appropriate selection of chemotherapy before and after surgery offers patients the best chances of prolonged survival and, potentially, cure.

An important consideration when evaluating these results is that, currently, only retrospective studies using registry datasets or small single-center cohorts have evaluated prognostic factors for LTS after resection of PDAC. Our results provide pooled estimates of these associations, incorporating the complete volume of true follow-up data available in the literature. We demonstrate the clinically significant finding that no given clinicopathologic feature is preclusive of LTS after surgery. The clinical implications of this finding are that surgeons may consider resection in patients despite the presence of poor prognostic factors, and that new biomarkers are needed to improve patient selection. However, our review also highlights the limitations of the current body of evidence and the paucity of nonactuarial data on LTS in specific clinical populations, such as those treated with various contemporary neoadjuvant or adjuvant regimens. Our findings emphasize the need for global partnerships across high-volume centers for large prospective studies to identify causal factors and independent predictors of LTS. This meta-analysis may act as a baseline consolidation of the current data for these future studies.

Several limitations should be considered when interpreting these data. First, several articles did not report complete data. In such cases, efforts were made to find corresponding full-text articles or obtain missing data from authors. Furthermore, the statistics extracted from articles were often formatted and reported inconsistently, requiring the data to be cleaned and restructured before analysis could be performed. Valid statistical methods, that have been indicated wherever used, were employed to make the data uniform and amenable to analysis. Second, included articles span more than 25 years, during which advances in the treatment of PDAC have occurred. However, given that the long-term prognosis of the disease remains dismal, reviewing all the available literature was deemed a priority over using more recent findings.

A strength of our review is that no estimates of LTS from survival analysis methods were used in our analysis and only actual patients surviving at least 5 years were included as LTS. By pooling together data from across studies in the literature that have investigated actual LTS after resection of PDAC, we have achieved adequate statistical power to estimate the effect sizes of these associations while excluding actuarial data. Moreover, since we have only analyzed patients who underwent resection, the patients included in our analysis were definitively diagnosed with PDAC based on surgical pathology. As such, the reliability of the data, such as the accuracy of tumor grading and the exclusion of other pancreatic neoplasms (such as neuroendocrine tumors or cystic neoplasms), is appropriate.

## Conclusions

While there appears to be evidence of a complex interplay between risk, tumor biology, patient characteristics, and management related factors, no single reviewed parameter can solely predict LTS after the resection of PDAC. Our results accurately quantify the associations between various clinicopathologic factors and true LTS in resected PDAC patients. However, further research is needed to identify causal factors involved in determining LTS in PDAC from these associations and stratify results to apply to specific clinical populations.

### Supplementary Information

Below is the link to the electronic supplementary material.

Supplementary file1 (DOCX 4820 kb)

## Data Availability

The data that support the findings of this study were sourced directly from the published studies included in this systematic review and meta-analysis.

## Availability of Protocol

While a review protocol was not prepared, our study was prospectively registered with PROSPERO (CRD42022346060).

## Prior Presentation Information

This study was accepted for poster presentation at the 2023 American Society of Clinical Oncology (ASCO) Annual Meeting.

## References

[1] Pancreatic Cancer—Cancer Stat Facts. Accessed 11 Oct 2022. https://seer.cancer.gov/statfacts/html/pancreas.html

[2] Principe DR, Underwood PW, Korc M, Trevino JG, Munshi HG, Rana A (2021). The current treatment paradigm for pancreatic ductal adenocarcinoma and barriers to therapeutic efficacy. Front Oncol..

[3] Wang Z, Li Y, Zhan S (2019). SMAD4 Y353C promotes the progression of PDAC. BMC Cancer..

[4] Groot VP, Rezaee N, Wu W (2018). Patterns, timing, and predictors of recurrence following pancreatectomy for pancreatic ductal adenocarcinoma. Ann Surg..

[5] Sohal DPS, Kennedy EB, Cinar P (2020). Metastatic pancreatic cancer: ASCO guideline update. J Clin Oncol..

[6] Jain T, Dudeja V (2021). The war against pancreatic cancer in 2020—advances on all fronts. Nat Rev Gastroenterol Hepatol..

[7] Bengtsson A, Andersson R, Ansari D (2020). The actual 5-year survivors of pancreatic ductal adenocarcinoma based on real-world data. Sci Rep..

[8] Moons KGM, Royston P, Vergouwe Y, Grobbee DE, Altman DG (2009). Prognosis and prognostic research: what, why, and how?. BMJ..

[9] Tonini V, Zanni M (2021). Pancreatic cancer in 2021: What you need to know to win. World J Gastroenterol..

[10] Wan X, Wang W, Liu J, Tong T (2014). Estimating the sample mean and standard deviation from the sample size, median, range and/or interquartile range. BMC Med Res Methodol..

[11] Wei JJ, Lin EX, Shi JD (2021). Meta-analysis with zero-event studies: a comparative study with application to COVID-19 data. Mil Med Res..

[12] Kuss O (2015). Statistical methods for meta-analyses including information from studies without any events—add nothing to nothing and succeed nevertheless. Stat Med..

[13] Xu C, Li L, Lin L (2020). Exclusion of studies with no events in both arms in meta-analysis impacted the conclusions. J Clin Epidemiol..

[14] Guyatt GH, Oxman AD, Vist GE (2008). GRADE: an emerging consensus on rating quality of evidence and strength of recommendations. BMJ..

[15] Belfiori G, Crippa S, Francesca A (2021). Long-term survivors after upfront resection for pancreatic ductal adenocarcinoma: an actual 5-year analysis of disease-specific and post-recurrence survival. Ann Surg Oncol..

[16] Benassai G, Mastrorilli M, Quarto G (2000). Factors influencing survival after resection for ductal adenocarcinoma of the head of the pancreas. J Surg Oncol..

[17] Conlon KCMD, Klimstra DSMD, Brennan MFMD, Brennan MF (1996). Long-term survival after curative resection for pancreatic ductal adenocarcinoma: Clinicopathologic analysis of 5-year survivors. Ann Surg..

[18] Dal Molin M, Zhang M, De Wilde RF (2015). Very long-term survival following resection for pancreatic cancer is not explained by commonly mutated genes: results of whole-exome sequencing analysis. Clin Cancer Res..

[19] Delcore R, Rodriguez FJ, Forster J, Hermreck AS, Thomas JH (1996). Significance of lymph node metastases in patients with pancreatic cancer undergoing curative resection. Am J Surg..

[20] Dusch N, Weiss C, Ströbel P, Kienle P, Post S, Niedergethmann M (2014). Factors predicting long-term survival following pancreatic resection for ductal adenocarcinoma of the pancreas: 40 years of experience. J Gastrointest Surg..

[21] Ferrone CR, Pieretti-Vanmarcke R, Bloom JP (2012). Pancreatic ductal adenocarcinoma: long-term survival does not equal cure. Surgery..

[22] Fukushima N, Sakamoto M, Mukai K (2001). Intraductal papillary components in invasive ductal carcinoma of the pancreas are associated with long-term survival of patients. Hum Pathol..

[23] Holm M, Saraswat M, Joenväärä S, Seppänen H, Renkonen R, Haglund C (2020). Label-free proteomics reveals serum proteins whose levels differ between pancreatic ductal adenocarcinoma patients with short or long survival. Tumor Biol..

[24] Han SH, Heo JS, Choi SH (2017). Actual long-term outcome of T1 and T2 pancreatic ductal adenocarcinoma after surgical resection. Int J Surg..

[25] Kardosh A, Lichtensztajn DY, Gubens MA, Kunz PL, Fisher GA, Clarke CA (2018). Long-term survivors of pancreatic cancer: a California population-based study. Pancreas..

[26] Katz MHG, Wang H, Fleming JB (2009). Long-term survival after multidisciplinary management of resected pancreatic adenocarcinoma. Ann Surg Oncol..

[27] Kimura K, Amano R, Nakata B (2014). Clinical and pathological features of five-year survivors after pancreatectomy for pancreatic adenocarcinoma. World J Surg Oncol..

[28] Luu AM, Braumann C, Belyaev O (2021). Long-term survival after pancreaticoduodenectomy in patients with ductal adenocarcinoma of the pancreatic head. Hepatobil Pancreatic Dis Int..

[29] Nakagawa K, Akahori T, Nishiwada S (2018). Prognostic factors for actual long-term survival in the era of multidisciplinary treatment for pancreatic ductal adenocarcinoma. Langenbecks Arch Surg..

[30] Nakagawa N, Yamada S, Sonohara F (2020). Clinical implications of naples prognostic score in patients with resected pancreatic cancer. Ann Surg Oncol..

[31] Nakano Y, Kitago M, Shinoda M (2017). Clinical predictive factors of long-term survival after curative resection of pancreatic cancer: a retrospective study. Cancer Med..

[32] Paniccia A, Hosokawa P, Henderson W (2015). Characteristics of 10-year survivors of pancreatic ductal adenocarcinoma. JAMA Surg..

[33] Picozzi VJ, Oh SY, Edwards A (2017). Five-year actual overall survival in resected pancreatic cancer: a contemporary single-institution experience from a multidisciplinary perspective. Ann Surg Oncol..

[34] Sadozai H, Acharjee A, Eppenberger-Castori S (2021). Distinct stromal and immune features collectively contribute to long-term survival in pancreatic cancer. Front Immunol..

[35] Schnelldorfer T, Ware AL, Sarr MG (2008). Long-term survival after pancreatoduodenectomy for pancreatic adenocarcinoma is cure possible?. Ann Surg..

[36] Shimada K, Sakamoto Y, Nara S, Esaki M, Kosuge T, Hiraoka N (2010). Analysis of 5-year survivors after a macroscopic curative pancreatectomy for invasive ductal adenocarcinoma. World J Surg..

[37] Shin SH, Kim SC, Hong SM (2014). Can statistically determined prognostic factors predict the long-term survival of patients with pancreatic ductal adenocarcinoma following surgical resection? Clinicopathological analysis of 82 long-term survivors. Pancreas..

[38] Sinn M, Striefler JK, Sinn BV (2013). Does long-term survival in patients with pancreatic cancer really exist?—Results from the CONKO-001 study. J Surg Oncol..

[39] Yamamoto T, Yagi S, Kinoshita H (2015). Long-term survival after resection of pancreatic cancer: a single-center retrospective analysis. World J Gastroenterol..

[40] Yoon KW, Heo JS, Choi DW, Choi SH (2011). Factors affecting long-term survival after surgical resection of pancreatic ductal adenocarcinoma. J Korean Surg Soc..

[41] Burgdorf SK, Storkholm JH, Chen IM, Hansen CP (2021). Postoperative and long-term survival in relation to life-expectancy after pancreatic surgery in elderly patients (cohort study). Ann Med Surg..

[42] Hartwig W, Strobel O, Hinz U (2013). CA19-9 in potentially resectable pancreatic cancer: perspective to adjust surgical and perioperative therapy. Ann Surg Oncol..

[43] Huhta H, Nortunen M, Meriläinen S, Helminen O, Kauppila JH (2022). Hospital volume and outcomes of pancreatic cancer: a Finnish population-based nationwide study. HPB..

[44] Kang JS, Jang JY, Kwon W, Han Y, Kim SW (2017). Clinicopathologic and survival differences in younger patients with pancreatic ductal adenocarcinoma—a propensity score-matched comparative analysis. Pancreatology..

[45] Shirai Y, Shiba H, Horiuchi T (2016). Assessment of surgical outcome after pancreatic resection in extremely elderly patients. Anticancer Res..

[46] Sugiura T, Okamura Y, Ito T, Yamamoto Y, Ashida R, Uesaka K (2017). Impact of patient age on the postoperative survival in pancreatic head cancer. Ann Surg Oncol..

[47] Natale CA, Li J, Pitarresi JR (2020). Pharmacologic activation of the G protein-coupled estrogen receptor inhibits pancreatic ductal adenocarcinoma. Cell Mol Gastroenterol Hepatol..

[48] Value of preoperative serum CA 19-9 levels in predicting resectability for pancreatic cancer | CJS. Accessed 15 March 2023. https://www.canjsurg.ca/content/49/4/241.long PMC3207573 16948881

[49] Bergquist JR, Puig CA, Shubert CR (2016). Carbohydrate antigen 19–9 elevation in anatomically resectable, early stage pancreatic cancer is independently associated with decreased overall survival and an indication for neoadjuvant therapy: A national cancer database study. J Am Coll Surg..

[50] Fujioka S, Misawa T, Okamoto T (2007). Preoperative serum carcinoembryonic antigen and carbohydrate antigen 19–9 levels for the evaluation of curability and resectability in patients with pancreatic adenocarcinoma. J Hepatobiliary Pancreat Surg..

[51] Karachristos A, Scarmeas N, Hoffman JP (2005). CA 19–9 levels predict results of staging laparoscopy in pancreatic cancer. J Gastrointest Surg..

[52] Maithel SK, Maloney S, Winston C (2008). Preoperative CA 19–9 and the yield of staging laparoscopy in patients with radiographically resectable pancreatic adenocarcinoma. Ann Surg Oncol..

[53] Maisey NR, Norman AR, Hill A, Massey A, Oates J, Cunningham D (2005). CA19-9 as a prognostic factor in inoperable pancreatic cancer: the implication for clinical trials. Br J Cancer..

[54] Clinical manifestations, diagnosis, and staging of exocrine pancreatic cancer - UpToDate. Accessed 15 March 2023. https://www.uptodate.com/contents/clinical-manifestations-diagnosis-and-staging-of-exocrine-pancreatic-cancer?search=CA-199&source=search_result&selectedTitle=1~63&usage_type=default&display_rank=1#H1620046

[55] Abdel-Misih SRZ, Hatzaras I, Schmidt C (2011). Failure of normalization of CA19-9 following resection for pancreatic cancer is tantamount to metastatic disease. Ann Surg Oncol..

[56] Kleeff J, Korc M, Apte M (2016). 2016 Pancreatic cancer. Nat Rev Dis Primers..

[57] Sadozai H, Acharjee A, Eppenberger-Castori S (2021). Distinct stromal and immune features collectively contribute to long-term survival in pancreatic cancer. Front Immunol..

[58] Delvecchio FR, Fincham REA, Spear S (2021). Pancreatic cancer chemotherapy is potentiated by induction of tertiary lymphoid structures in mice. Cell Mol Gastroenterol Hepatol..

[59] Guillermet-Guibert J, Davenne L, Pchejetski D (2009). Targeting the sphingolipid metabolism to defeat pancreatic cancer cell resistance to the chemotherapeutic gemcitabine drug. Mol Cancer Ther..

[60] Kleeff J, Korc M, Apte M (2016). 2016 Pancreatic cancer. Nat Rev Disease Primers..

[61] Springfeld C, Neoptolemos JP (2022). The role of neoadjuvant therapy for resectable pancreatic cancer remains uncertain. Nat Rev Clin Oncol..

[62] Oba A, Ho F, Bao QR, Al-Musawi MH, Schulick RD, Del Chiaro M (2020). Neoadjuvant treatment in pancreatic cancer. Front Oncol..

[63] Michelakos T, Pergolini I, del Castillo CF (2019). Predictors of resectability and survival in patients with borderline and locally advanced pancreatic cancer who underwent neoadjuvant treatment with FOLFIRINOX. Ann Surg..

[64] van Dam JL, Janssen QP, Besselink MG (2022). Neoadjuvant therapy or upfront surgery for resectable and borderline resectable pancreatic cancer: A meta-analysis of randomised controlled trials. Eur J Cancer..

