# Supplementary material for: Predictors for Long-Term Survival After Resection of Pancreatic Ductal Adenocarcinoma: A Systematic Review and Meta-Analysis
Source: Ann Surg Oncol. 2024 May 6;31(7):4673–87. doi: 10.1245/s10434-024-15281-1 (PMC11164751; doi:10.1245/s10434-024-15281-1)
Supplement: Supplementary file 1 — Supplementary file1 (DOCX 4820 kb) [file 10434_2024_15281_MOESM1_ESM.docx]

**Predictors for Long-Term Survival after Resection of Pancreatic Ductal Adenocarcinoma: A Systematic Review and Meta-Analysis**

**Supplementary Material**

*Supplementary Section 1:* PRISMA Checklist (2020)

*Supplementary Section 2:* Complete Search Strings

*Supplementary Section 3:* Estimations from Non-Parametrically Distributed Data

*Supplementary Section 4:* Study Quality Assessment

*Supplementary Section 5:* Extended Results

*Supplementary Section 6:* Forest Plots

*Supplementary Section 7:* GRADE Evidence Profile

**Section 1: PRISMA Checklist (2020)**

| **Section and Topic** | **Item #** | **Checklist item** | **Location where item is reported** |
| --- | --- | --- | --- |
| **TITLE** | | |  |
| Title | 1 | Identify the report as a systematic review. | 1 |
| **ABSTRACT** | | |  |
| Abstract | 2 | See the PRISMA 2020 for Abstracts checklist. | 3 |
| **INTRODUCTION** | | |  |
| Rationale | 3 | Describe the rationale for the review in the context of existing knowledge. | 4 |
| Objectives | 4 | Provide an explicit statement of the objective(s) or question(s) the review addresses. | 4 |
| **METHODS** | | |  |
| Eligibility criteria | 5 | Specify the inclusion and exclusion criteria for the review and how studies were grouped for the syntheses. | 5 |
| Information sources | 6 | Specify all databases, registers, websites, organisations, reference lists and other sources searched or consulted to identify studies. Specify the date when each source was last searched or consulted. | 5 |
| Search strategy | 7 | Present the full search strategies for all databases, registers and websites, including any filters and limits used. | 5, Supplementary Section 2 |
| Selection process | 8 | Specify the methods used to decide whether a study met the inclusion criteria of the review, including how many reviewers screened each record and each report retrieved, whether they worked independently, and if applicable, details of automation tools used in the process. | 5, 6 |
| Data collection process | 9 | Specify the methods used to collect data from reports, including how many reviewers collected data from each report, whether they worked independently, any processes for obtaining or confirming data from study investigators, and if applicable, details of automation tools used in the process. | 5, 6, 7 |
| Data items | 10a | List and define all outcomes for which data were sought. Specify whether all results that were compatible with each outcome domain in each study were sought (e.g. for all measures, time points, analyses), and if not, the methods used to decide which results to collect. | 5, 6 |
|  | 10b | List and define all other variables for which data were sought (e.g. participant and intervention characteristics, funding sources). Describe any assumptions made about any missing or unclear information. | 5, 6 |
| Study risk of bias assessment | 11 | Specify the methods used to assess risk of bias in the included studies, including details of the tool(s) used, how many reviewers assessed each study and whether they worked independently, and if applicable, details of automation tools used in the process. | 8 |
| Effect measures | 12 | Specify for each outcome the effect measure(s) (e.g. risk ratio, mean difference) used in the synthesis or presentation of results. | 8, 9 |
| Synthesis methods | 13a | Describe the processes used to decide which studies were eligible for each synthesis (e.g. tabulating the study intervention characteristics and comparing against the planned groups for each synthesis (item #5)). | 8, 9 |
|  | 13b | Describe any methods required to prepare the data for presentation or synthesis, such as handling of missing summary statistics, or data conversions. | 8, 9 |
|  | 13c | Describe any methods used to tabulate or visually display results of individual studies and syntheses. | 8, 9 |
|  | 13d | Describe any methods used to synthesize results and provide a rationale for the choice(s). If meta-analysis was performed, describe the model(s), method(s) to identify the presence and extent of statistical heterogeneity, and software package(s) used. | 8, 9 |
|  | 13e | Describe any methods used to explore possible causes of heterogeneity among study results (e.g. subgroup analysis, meta-regression). | 8, 9 |
|  | 13f | Describe any sensitivity analyses conducted to assess robustness of the synthesized results. | 8, 9 |
| Reporting bias assessment | 14 | Describe any methods used to assess risk of bias due to missing results in a synthesis (arising from reporting biases). | 8 |
| Certainty assessment | 15 | Describe any methods used to assess certainty (or confidence) in the body of evidence for an outcome. | 8 |
| **RESULTS** | | |  |
| Study selection | 16a | Describe the results of the search and selection process, from the number of records identified in the search to the number of studies included in the review, ideally using a flow diagram. | 10 |
|  | 16b | Cite studies that might appear to meet the inclusion criteria, but which were excluded, and explain why they were excluded. | 10 |
| Study characteristics | 17 | Cite each included study and present its characteristics. | 10, Table 1 |
| Risk of bias in studies | 18 | Present assessments of risk of bias for each included study. | 10, Supplementary Section 4 |
| Results of individual studies | 19 | For all outcomes, present, for each study: (a) summary statistics for each group (where appropriate) and (b) an effect estimate and its precision (e.g. confidence/credible interval), ideally using structured tables or plots. | 10 – 18, Tables 2 and 3 |
| Results of syntheses | 20a | For each synthesis, briefly summarise the characteristics and risk of bias among contributing studies. | 10 – 18, Supplementary Section 5 |
|  | 20b | Present results of all statistical syntheses conducted. If meta-analysis was done, present for each the summary estimate and its precision (e.g. confidence/credible interval) and measures of statistical heterogeneity. If comparing groups, describe the direction of the effect. | 10 – 18, Supplementary Section 5, 6 |
|  | 20c | Present results of all investigations of possible causes of heterogeneity among study results. | 10 – 18, Tables 2 and 3 |
|  | 20d | Present results of all sensitivity analyses conducted to assess the robustness of the synthesized results. | Supplementary Section 5 |
| Reporting biases | 21 | Present assessments of risk of bias due to missing results (arising from reporting biases) for each synthesis assessed. | 10 |
| Certainty of evidence | 22 | Present assessments of certainty (or confidence) in the body of evidence for each outcome assessed. | 10, Tables 2 and 3 |
| **DISCUSSION** | | |  |
| Discussion | 23a | Provide a general interpretation of the results in the context of other evidence. | 19 – 24 |
|  | 23b | Discuss any limitations of the evidence included in the review. | 24 |
|  | 23c | Discuss any limitations of the review processes used. | 24 |
|  | 23d | Discuss implications of the results for practice, policy, and future research. | 19 – 25 |
| **OTHER INFORMATION** | | |  |
| Registration and protocol | 24a | Provide registration information for the review, including register name and registration number, or state that the review was not registered. | 5 |
|  | 24b | Indicate where the review protocol can be accessed, or state that a protocol was not prepared. | 26 |
|  | 24c | Describe and explain any amendments to information provided at registration or in the protocol. | N/A |
| Support | 25 | Describe sources of financial or non-financial support for the review, and the role of the funders or sponsors in the review. | 26 |
| Competing interests | 26 | Declare any competing interests of review authors. | 26 |
| Availability of data, code and other materials | 27 | Report which of the following are publicly available and where they can be found: template data collection forms; data extracted from included studies; data used for all analyses; analytic code; any other materials used in the review. | 26 |

*From:*  Page MJ, McKenzie JE, Bossuyt PM, Boutron I, Hoffmann TC, Mulrow CD, et al. The PRISMA 2020 statement: an updated guideline for reporting systematic reviews. BMJ 2021;372:n71. doi: 10.1136/bmj.n71

For more information, visit: <http://www.prisma-statement.org/>

**Section 2: Search Strings** *(searched in April 2022)*

**PubMed: 986 Articles**

("long term survival"[All Fields] OR "long-term survival"[All Fields]) AND ("carcinoma, pancreatic ductal"[MeSH Terms] OR "Pancreatic Cancer"[All Fields] OR "Pancreatic Adenocarcinoma"[All Fields] OR "PDAC"[All Fields])

**Embase: 2,337 Articles**

('long term survival'/exp OR 'long term survival' OR 'long-term survival'/exp OR 'long-term survival') AND ('carcinoma, pancreatic ductal'/exp OR 'carcinoma, pancreatic ductal' OR 'pancreatic cancer'/exp OR 'pancreatic cancer' OR 'pancreatic adenocarcinoma'/exp OR 'pancreatic adenocarcinoma' OR pdac)

**Scopus: 1,263 Articles**

TITLE-ABS-KEY ( ( ( "Long Term Survival" ) OR ( "Long-Term Survival" ) ) AND ( ( "Carcinoma, Pancreatic Ductal" ) OR ( "Pancreatic Cancer" ) OR ( "Pancreatic Adenocarcinoma" ) OR ( pdac ) ) )

**Cochrane CENTRAL: 67 Articles**

((“Long Term Survival”) OR (“Long-Term Survival”)) AND (("Carcinoma, Pancreatic Ductal"[Mesh]) OR (“Pancreatic Cancer”) OR (“Pancreatic Adenocarcinoma”) OR (PDAC))

**Section 3: Estimations from Non-Parametrically Distributed Data**

**Age**

**Means and SD’s that have been calculated from Medians, Ranges, and/or IQR’s*

****Mean, Median, and Range was reported so an estimation of the standard deviation was made, but the reported mean is used*

| **Study** | **Sample Size** | | **LTS** | | **Non-LTS** | |
| --- | --- | --- | --- | --- | --- | --- |
|  | **LTS** | **Non-LTS** | **Mean** | **SD** | **Mean** | **SD** |
| Sadozai et al. 2021* | 25 | 87 | 68.33 | 11.005 | 65 | 9.046 |
| Nakagawa et al. 2018* | 38 | 90 | 68 | 6.554 | 63.25 | 9.955 |
| Kardosh et al. 2018* | 742 | 5682 | 60.75 | 10.984 | 65.5 | 11.367 |
| Nakano et al. 2017* | 38 | 133 | 66.5 | 6.086 | 65.75 | 7.121 |
| Paniccia et al. 2015 | 431 | 10650 | 61.8 | 10.6 | 65.6 | 10.7 |
| Yamamoto et al. 2015 | 20 | 76 | 65.5 | 9.4 | 67.2 | 7.9 |
| Dal Molin et al. 2015 | 35 | 226 | 59.1 | 10.72 | 65.7 | 11.19 |
| Kimura et al. 2014* | 18 | 129 | 63 | 8.233 | 63 | 10.049 |
| Yoon et al. 2011 | 19 | 145 | 56.26 | 9.03 | 59.22 | 10.03 |
| Luu et al. 2021* | 34 | 133 | 64.67 | 11.609 | 65.33 | 11.99 |
| Katz et al. 2009*** | 88 | 241 | 62 | 11.21 | 63 | 7.691 |

**BMI**

**Means and SD’s that have been calculated from Medians, Ranges, and/or IQR’s*

| **Study** | **Sample Size** | | **LTS** | | **Non-LTS** | |
| --- | --- | --- | --- | --- | --- | --- |
|  | **LTS** | **Non-LTS** | **Mean** | **SD** | **Mean** | **SD** |
| Nakano et al. 2017* | 38 | 133 | 26 | 5.524 | 24.25 | 3.387 |
| Nakagawa et al. 2018* | 38 | 90 | 22.25 | 3.028 | 21.75 | 2.641 |
| Luu et al. 2021* | 34 | 133 | 25.5 | 1.434 | 24 | 0.769 |

**Tumor Size**

**Means and SD’s that have been calculated from Medians, Ranges, and/or IQR’s*

***Mean and Range was reported, so an estimation of the standard deviation was made assuming that the mean is equal to the median given that the algorithm requires a median and not a mean*

****Mean, Median, and Range was reported so an estimation of the standard deviation was made, but the reported mean is used*

| **Study** | **Sample Size** | | **LTS** | | **Non-LTS** | |
| --- | --- | --- | --- | --- | --- | --- |
|  | **LTS** | **Non-LTS** | **Mean** | **SD** | **Mean** | **SD** |
| Sadozai et al. 2021* | 25 | 87 | 2.833 | 0.393 | 3.167 | 1.131 |
| Dal Molin et al. 2015 | 35 | 226 | 2.8 | 1.18 | 3.1 | 1.61 |
| Dusch et al. 2014** | 69 | 291 | 2.40 | 0.334 | 6.30 | 0.980 |
| Kimura et al. 2014* | 18 | 129 | 3.275 | 1.180 | 6.55 | 3.247 |
| Katz et al. 2009*** | 88 | 241 | 2.8 | 1.569 | 3.1 | 2.289 |
| Schnelldorfer et al. 2008 | 62 | 295 | 2.7 | 1 | 3.3 | 1.2 |

**Preoperative Bilirubin**

**Means and SD’s that have been calculated from Medians, Ranges, and/or IQR’s*

***Mean and Range was reported, so an estimation of the standard deviation was made assuming that the mean is equal to the median given that the algorithm requires a median and not a mean*

| **Study** | **Sample Size** | | **LTS** | | **Non-LTS** | |
| --- | --- | --- | --- | --- | --- | --- |
|  | **LTS** | **Non-LTS** | **Mean** | **SD** | **Mean** | **SD** |
| Nakano et al. 2017* | 38 | 133 | 1.525 | 0.913 | 2.175 | 1.174 |
| Dusch et al. 2014** | 69 | 291 | 6.725 | 4.673 | 9.634 | 5.401 |

**Preoperative Albumin**

**Means and SD’s that have been calculated from Medians, Ranges, and/or IQR’s*

***Mean and Range was reported, so an estimation of the standard deviation was made assuming that the mean is equal to the median given that the algorithm requires a median and not a mean*

| **Study** | **Sample Size** | | **LTS** | | **Non-LTS** | |
| --- | --- | --- | --- | --- | --- | --- |
|  | **LTS** | **Non-LTS** | **Mean** | **SD** | **Mean** | **SD** |
| Nakano et al. 2017* | 38 | 133 | 3.825 | 0.492 | 3.775 | 0.404 |
| Dusch et al. 2014** | 69 | 291 | 4.03 | 0.588 | 3.683 | 0.720 |
| Yoon et al. 2011 | 19 | 145 | 4.06 | 0.49 | 3.80 | 0.55 |

**Preoperative CA19-9**

**Means and SD’s that have been calculated from Medians, Ranges, and/or IQR’s*

***Mean and Range was reported, so an estimation of the standard deviation was made assuming that the mean is equal to the median given that the algorithm requires a median and not a mean*

| **Study** | **Sample Size** | | **LTS** | | **Non-LTS** | |
| --- | --- | --- | --- | --- | --- | --- |
|  | **LTS** | **Non-LTS** | **Mean** | **SD** | **Mean** | **SD** |
| Sadozai et al. 2021* | 25 | 87 | 331.33 | 601.38 | 2415 | 5129.96 |
| Nakagawa et al. 2018* | 38 | 90 | 220.25 | 172.155 | 1749 | 1372.546 |
| Nakano et al. 2017* | 38 | 133 | 1832.75 | 1694.526 | 2249.25 | 1697.363 |
| Yamamoto et al. 2015* | 20 | 76 | 156.67 | 273.661 | 285.33 | 412.984 |
| Dusch et al. 2014** | 69 | 291 | 3041.75 | 2542.312 | 20139.55 | 14048.9 |
| Luu et al. 2021* | 34 | 133 | 82.25 | 34.189 | 262 | 133.187 |

**Preoperative CEA**

**Means and SD’s that have been calculated from Medians, Ranges, and/or IQR’s*

***Mean and Range was reported, so an estimation of the standard deviation was made assuming that the mean is equal to the median given that the algorithm requires a median and not a mean*

| **Study** | **Sample Size** | | **LTS** | | **Non-LTS** | |
| --- | --- | --- | --- | --- | --- | --- |
|  | **LTS** | **Non-LTS** | **Mean** | **SD** | **Mean** | **SD** |
| Nakano et al. 2017* | 38 | 133 | 14.65 | 12.125 | 16.95 | 11.548 |
| Yamamoto et al. 2015* | 20 | 76 | 2 | 1.117 | 3.33 | 2.872 |
| Dusch et al. 2014** | 69 | 291 | 1.9 | 10.488 | 2.7 | 166.67 |
| Luu et al. 2021* | 34 | 133 | 2.995 | 0.569 | 3.475 | 0.789 |

**Operative Blood Loss (MD)**

**Means and SD’s that have been calculated from Medians, Ranges, and/or IQR’s*

***Mean and Range was reported, so an estimation of the standard deviation was made assuming that the mean is equal to the median given that the algorithm requires a median and not a mean*

****Mean, Median, and Range was reported so an estimation of the standard deviation was made, but the reported mean is used*

| **Study** | **Sample Size** | | **LTS** | | **Non-LTS** | |
| --- | --- | --- | --- | --- | --- | --- |
|  | **LTS** | **Non-LTS** | **Mean** | **SD** | **Mean** | **SD** |
| Nakagawa et al. 2018* | 38 | 90 | 953.5 | 568.35 | 1257.5 | 715.12 |
| Nakano et al. 2017* | 38 | 113 | 950.31 | 609.3 | 1719.83 | 1048.75 |
| Dusch et al. 2014** | 65 | 291 | 1396.12 | 747.53 | 2129.67 | 1160 |
| Katz et al. 2009*** | 88 | 241 | 1057 | 708.296 | 1374 | 3201.53 |

**Operative Time (Minutes)**

**Means and SD’s that have been calculated from Medians, Ranges, and/or IQR’s*

***Mean and Range was reported, so an estimation of the standard deviation was made assuming that the mean is equal to the median given that the algorithm requires a median and not a mean*

| **Study** | **Sample Size** | | **LTS** | | **Non-LTS** | |
| --- | --- | --- | --- | --- | --- | --- |
|  | **LTS** | **Non-LTS** | **Mean** | **SD** | **Mean** | **SD** |
| Nakagawa et al. 2018* | 38 | 90 | 338.25 | 101.358 | 349.25 | 100.564 |
| Nakano et al. 2017* | 38 | 113 | 622.5 | 180.946 | 633.5 | 193.979 |
| Dusch et al. 2014** | 65 | 291 | 383.5 | 93.719 | 441.25 | 133.934 |
| Yoon et al. 2011 | 19 | 145 | 312.63 | 101.75 | 344.62 | 101.70 |

**Hospital Stay (Days)**

**Means and SD’s that have been calculated from Medians, Ranges, and/or IQR’s*

****Mean, Median, and Range was reported so an estimation of the standard deviation was made, but the reported mean is used*

| **Study** | **Sample Size** | | **LTS** | | **Non-LTS** | |
| --- | --- | --- | --- | --- | --- | --- |
|  | **LTS** | **Non-LTS** | **Mean** | **SD** | **Mean** | **SD** |
| Luu et al. 2021* | 34 | 133 | 16.7 | 2.582 | 16 | 1.540 |
| Yoon et al. 2011 | 19 | 145 | 19.95 | 8.52 | 23.02 | 19.66 |
| Katz et al. 2009*** | 88 | 241 | 13 | 7.134 | 15 | 18.422 |

**Section 4: Study Quality Assessment**

**Supplementary Table:** Quality assessment of included observational studies using the Newcastle-Ottawa Scale

| Study | Year | NOS Domain | | | | | | | | | | |
| --- | --- | --- | --- | --- | --- | --- | --- | --- | --- | --- | --- | --- |
|  |  | **Selection** | | | | **Comparability** | | **Outcome/Exposure** | | | **Total Score** | **Study Quality** |
|  |  | **S1** | **S2** | **S3** | **S4** | **C1** | **C2** | **O1** | **O2** | **O3** |  |  |
| Belfiori et al. | 2021 | ***** | ***** | ***** | ***** | ***** | ***** | ***** | ***** | ***** | 9 | Good |
| Benassai et al. | 2000 |  | * |  |  |  |  | * |  |  | 2 | Poor |
| Conlon et al. | 1996 |  | * | * | * | * |  | * | * | * | 7 | Good |
| Dal Molin et al. | 2015 | * | * | * | * |  | * | * | * | * | 8 | Good |
| Delcore et al. | 1996 |  | * | * | * | * | * | * | * |  | 7 | Good |
| Dusch et al | 2014 |  | * | * | * | * | * | * | * | * | 8 | Good |
| Ferrone et al. | 2012 | * | * | * | * | * | * | * | * |  | 8 | Good |
| Fukushima et al. | 2001 | * | * | * | * | * | * | * | * | * | 9 | Good |
| Han et al. | 2017 | * | * | * | * | * | * | * | * | * | 9 | Good |
| Holm et al. | 2020 | * | * | * | * |  | * | * | * | * | 8 | Good |
| Kardosh et al. | 2018 | * | * | * | * | * | * | * | * | * | 9 | Good |
| Katz et al. | 2009 | * | * | * | * | * | * | * | * | * | 9 | Good |
| Kimura et al. | 2014 |  | * | * | * | * | * | * | * | * | 8 | Good |
| Luu et al. | 2021 |  | * | * | * | * | * | * | * |  | 7 | Good |
| Nakagawa et al. | 2018 | * | * | * | * | * | * | * | * | * | 9 | Good |
| Nakano et al. (2017) | 2017 | * | * | * | * | * | * | * | * |  | 8 | Good |
| Paniccia et al. | 2015 | * | * | * | * | * | * | * | * | * | 9 | Good |
| Picozzi et al. | 2017 | * | * | * | * | * | * | * | * | * | 9 | Good |
| Sadozai et al. | 2021 | * | * | * | * | * | * | * | * | * | 9 | Good |
| Schnelldorfer et al. | 2008 | * | * | * | * | * | * | * | * | * | 9 | Good |
| Shimada et al. | 2010 |  | * | * | * | * | * | * | * | * | 9 | Good |
| Shin et al. | 2014 | * | * | * | * |  | * | * | * | * | 8 | Good |
| Sinn et al. | 2013 | * | * | * | * | * | * | * | * |  | 8 | Good |
| Yamamoto et al. | 2015 | * | * | * | * | * | * | * | * | * | 9 | Good |
| Yoon et al. | 2011 |  | * | * | * | * | * | * | * | * | 9 | Good |
| Bengtsson et al. | 2020 | * | * | * | * | * | * | * | * | * | 9 | Good |
| Burgdorf et al. | 2021 | * | * | * | * | * | * | * | * | * | 9 | Good |
| Hartwig et al. | 2013 | * |  | * | * | * | * | * | * | * | 8 | Good |
| Huhta et al. | 2021 | * | * | * | * |  | * | * |  |  | 6 | Fair |
| Kang et al. | 2017 | * | * | * | * | * | * | * | * | * | 9 | Good |
| Nakagawa et al. | 2020 | * | * | * | * | * |  | * | * | * | 8 | Good |
| Shirai et al. | 2016 | * | * | * | * | * | * | * | * |  | 8 | Good |
| Sugiura et al. | 2017 | * | * | * | * |  | * | * |  |  | 6 | Fair |

**Section 5: Extended Results**

**Patient Factors Associated with Long-Term Survival**

**Age**

Pooling 11 studies’ data showed no significant difference in the age of patients who achieved long-term survival after resection and those who did not (mean difference: -1.31, 95% CI: -3.18, 0.56). However, a sensitivity analysis removing the results of Nakagawa et al’s study – the only study showing long-term survivors to be significantly older than other patients - from the analysis resulted in a significant mean difference of –2.08 (95% CI: -3.68, -0.47). However, no clear methodological difference that could explain why their results deviated from the general trend of the studies analyzed was identified (and hence this sensitivity analysis is not included in the main manuscript).

Shimada et al. dichotomized age at a cutoff value of 64 years and found no significant association with long-term survival. Picozzi et al. obtained a similar result having set 65 years as their cutoff.

Sinn et al. categorized patients into ordinal groups of increasing age and found no significant association between age and LTS.

Bengtsson et al. found a significant association between age and long-term survival on multivariate analysis (OR [95% CI]: 0.988 (0.982–0.994)).

**Biological Sex**

20 studies were meta-analyzed and a significant association between female biological sex and long-term survival was found, with an odds ratio of 1.29 (95% CI: 1.01 - 1.64).

Bengtsson et al. also found female biological sex to be independently associated with LTS, with an odds ratio of 1.13 (1.01–1.27) on multivariate analysis.

**BMI**

3 studies reported poolable data on the association between BMI and long-term survival. Long-term survivors had a higher mean BMI in all three studies and meta-analysis showed a statistically significant overall mean difference of 1.27 (kg/sq.m) (95% CI: 0.61,1.92).

2 additional studies, Yoon et al. And Picozzi et al., reported BMI related results that were not meta-analyzable. Yoon et al. sorted patients into normal BMI (<25) and obese categories while Picozzi et al. sorted patients into <24.9, 25.0-29.9, and >29.9 BMI categories. Neither study found a significant association between BMI and LTS in these analyses.

**Alcohol and Nicotine Use**

Data from Dusch et al. and Luu et al’s. studies were meta-analyzed to calculate the overall association between alcohol use by patients and achieving long-term survival. A null result was found, with an insignificant odds ratio of 0.39 (95% CI: 0.04,3.61).

Luu et al. also reported no significant association between LTS and nicotine use.

**Assorted Socioeconomic Determinants**

Kardosh et al. and Paniccia et al. defined LTS as survival beyond 10 years and investigated potential associations between various socioeconomic factors and LTS.

Neither study found significant associations between race and LTS in patients who underwent resection.

Kardosh et al. found that the patients from the 4^th^ and 5^th^ quintiles of socioeconomic status had a significantly higher likelihood of achieving LTS than others. However, Paniccia et al. found no association between income quartile and odds of surviving at least a decade.

Paniccia et al. also assessed the association between LTS and the percentage of people in patients’ area of residence with a high-school diploma, as a proxy for educational status. They found that patients from areas where over 86% of the community was at least high school educated had a significant and independent survival advantage. However, the distance of these areas of residence from the hospital, and whether they were rural, urban, or metropolitan settings, were not found to modify the odds of surviving long-term.

Paniccia et al. did not find private, Medicare, or Medicaid insurance to independently increase the odds of LTS when compared to uninsured patients.

**Preoperative Bilirubin**

3 studies provided results on the association of pre-operative bilirubin levels with the odds of achieving long-term survival after resection. All 3 studies reported statistically significant differences between long-term survivors and other patients.

Of these, two studies reported meta-analyzable data, and a mean difference of -1.69 (95% CI: -3.90, 0.51) was obtained.

**Presence of Biliary Stenting Pre-operatively**

3 studies reported meta-analyzable data on the association between pre-operative biliary stenting and long-term survival. An insignificant odds-ratio of 0.62 (95% CI: 0.24, 1.61) was obtained.

**Preoperative Albumin**

Meta-analysis of 3 studies showed a statistically significant mean difference of 0.22 (95% CI: 0.03,0.41), with LTS patients having higher pre-operative albumin levels.

**Preoperative CA19-9**

6 studies were pooled to show that pre-operative ca19-9 levels were significantly lower in LTS patients than others. The average pre-operative ca19-9 levels in LTS patients from these 6 studies ranged from 45 to 159 units/ml, while those of NLTS patients ranged from 88 to 380 units/ml. All 6 studies found lower pre-operative ca19-9 levels in LTS patients than in NLTS patients. However, upon conversion to mean and standard deviation datapoints, extreme heterogeneity was seen in the data when pooled. This occurred due to the extremely large ranges and inter-quartile ranges associated with the data points. The overall mean-difference obtained by meta-analyzing these values remained statistically significant and reflects the association between LTS and pre-operative ca19-9 levels, but the value and associated confidence interval are not meaningful as a result.

5 studies reported results on preoperative ca19-9 levels that were not meta-analyzable. Kimura et al. dichotomized pre-op ca19-9 at 40u/ml and reported an odds ratio of 5.02 (95% CI: 1.68-16.48). Yoon et al. and Shimada et al also found significant associations (p=0.001 and 0.0068) using thresholds of 37u/ml and 206 IU/dl. Hartwig et al. found a general trend of decreased LTS in stratified patients into multiple categories of ascending pre-op ca19-9 levels.

Sinn et al. found an insignificant association (p=0.36) between preoperative ca19-9 and survival having dichotomized at the threshold of 40u/ml.

Picozzi et al. found that there was no difference in odds of LTS in patients with post-operative ca19-9 levels above or below 100units/L.

**Preoperative CEA (Carcino-embryonic Antigen)**

Four studies were meta-analyzed to assess the differences in pre-operative CEA (in ng/mL) between LTS and non-LTS patients and a significant overall MD of -0.79 [-1.41, -0.17] was obtained.

Shimada et al. dichotomized patients into two groups using a threshold of 3.2IU/ml of pre-operative CEA and found no association with achieving long-term survival.

**Comorbidities**

The associations between long-term survival and various pre-operative comorbidities were reviewed.

Three studies’ data were pooled to compute an overall odds ratio of 0.70 (95% CI: 0.50, 0.99) for the presence of diabetes in long-term survivors. Kleef et al. comprehensively studied the association of diabetes and survival in pancreatic cancer. Their evidence highlighted a similar detrimental effect of diabetic status on patients’ prognoses and identified differences in the importance of tumor size and systemic effects in patients with insulin dependent and non-insulin dependent diabetes.

Dusch et al. and Luu et al. reported data on cardiovascular disease for which an overall odds ratio of 1.22 (95% CI: 0.74 - 2.02) was obtained.

Dusch et al. also reported data on the associations of LTS with numerous other comorbidities, such as hypertension, pulmonary disease, chronic pancreatitis, and the presence of abdominal or back pain, and other pathologic conditions. However, only the presence of pre-operative stenting was significantly associated with surviving beyond 5 years (as were pre-operative ASA scores and ca19-9 levels, for which we have meta-analyzed the reported data in the pooled analyses for these respective factors.)

Luu et al. reported no significant association between exocrine pancreatic insufficiency and LTS.

**Preoperative ASA classification and ECOG status**

An ASA classification of 3 or above was defined as an event and meta-analysis of data from 3 studies yielded an overall odds ratio 0.80 (95% CI: 0.42, 1.52), showing no significant association with long-term survival.

Yoon et al. did not report meta-analyzable data but, defining ASA scores >1 as events, also found no difference between LTS patients and those who did not achieve long-term survival.

Picozzi et al. compared the odds of LTS in patients with an ECOG score at or above 1 with other patients. Long-term survivors had significantly higher odds of a lower ECOG score (indicating superior health and functional status).

**Neutrophil Lymphocyte Ratio**

Nakano et al’s data showed a mean difference of -0.78 (95% CI: -1.36, -0.19) between LTS and NLTS patients; the latter had significantly higher NLRs on average.

Abe et al. also found that lower NLR was predictive of LTS having dichotomized it at a cut-off value of 2.2, with greater long-term survival in patients with lower NLRs.

**Tumor Factors Associated with Long-term Survival**

**Tumor Location**

Data were pooled from 12 studies to analyze the prognostic value of tumor location, comparing neoplasms occurring in the head of the pancreas with those occurring at other sites. An overall odds ratio of 0.96 (95% CI: 0.84,1.09) was computed, suggesting that tumor location is not a predictor of long-term survival based on the evidence from these articles. 2 of the studies included defined long-term survival as 10 years or greater, and two included studies had disproportionately large sample sizes. However, sensitivity analyses involving these studies did not significantly alter the calculated result.

Although not meta-analyzable, Bengtsson et al. reported results of univariable logistic regression analysis of the association between tumor location and long-term survival using data from 7564 patients who underwent resection. The odds ratios for the pancreatic body, tail, and other sites, using the pancreatic head as reference, were 1.33(95% CI: 1.04,1.70) for the body, 1.26 (95% CI: 1.02,1.55) for the tail, and 0.969(95% CI: 0.791,1.19) for other sites.

**Tumor Size**

16 of the included studies reported data on tumor size as a predictor of long-term survival.

6 articles provided data on tumor size that could be used to calculate a pooled mean difference. The point estimates of tumor size were lower in LTSs than NLTSs in all pooled studies but a statistically insignificant overall mean difference of -1.45 cm (95% CI: -3.19, 0.30) was obtained.

Other studies dichotomized tumor size but used differing cutoff sizes, preventing pooling of their results. 4 studies found significant associations between tumor size and LTS, while 4 studies did not. Paniccia et al. divided patients into three categories of <20mm, 20-40mm, and >40m sized tumors, and defined long-term survival as 10 years. Their data showed that using the >4cm category as a reference, the odds ratios for tumor size in long-term survivors were 1.2(0.9-1.5) for 20-40mm tumors and 1.7(1.2-2.5) for <20m tumors.

**Tumor Grade**

Tumor grade data were dichotomized and meta-analyzed in two alternate ways to compute LTS odds ratios – the results of both approaches are reported.

In the first approach – moderately, poorly, and undifferentiated tumors were compared as a group with well differentiated tumors. In this manner, data from 13 studies were pooled to compute an overall odds ratio of 0.40 (95% CI: 0.31,0.52).

In the second approach – poorly differentiated and anaplastic tumors were considered one category and well or moderately differentiated tumors another. Data from 19 studies were meta-analyzed, yielding an overall odds ratio of 0.57 (95% CI: 0.45,0.74).

2 studies reported results that could not be incorporated into either of these approaches. Using well-differentiated tumors as a reference, multivariate analysis by Bengtsson et al. found that long-term survivors had significantly lower odds of having higher grade tumors [moderately differentiated (OR = 0.590 (0.474–0.734); poorly differentiated/anaplastic (OR = 0.431 (0.343–0.542)]. However, Shimada et al. did not find that LTS was associated with the presence of a well-differentiated tumor as opposed to a moderate or poorly differentiated one.

**Pathologic T-Stage**

With events defined as a pathologic T-stage of 3 or above, meta-analysis of 13 studies’ data showed a significant negative association with long-term survival (odds ratio: 0.40, 95% CI: 0.31, 0.52).

Bengtsson et al. did not report poolable data, but also found that T-stages 3 and 4 were both independently associated with poorer chances of LTS.

**Pathologic M-Stage (presence of distant metastases)**

Pooling data from 4 studies, distant metastases were significantly associated with long-term survival. An odds ratio of 0.16 (95% CI: 0.06-0.38) was calculated.

Bengtsson at al’s. results could not be meta-analyzed, but also showed the presence of distant metastases to be significantly associated with decreased chances of LTS.

**Mutations**

The effects of 4 mutations, namely KRAS, TP53, SMAD4, and CDK2NA were reviewed but data were not poolable for meta-analysis.

Sadozai et al. reported data that were used to calculate odds ratios for KRAS, TP53, CDK2NA, and SMAD4 mutations, which were found to be 4.60 (95% CI: 0.57,36.86), 1.18 (95% CI: 0.46,3.04), 0.33 (95% CI: 0.07,1.55), and 0.54 (95% CI: 0.11,2.67) respectively. Thus, no conclusions can be drawn regarding the utility of detecting these mutations to help predict long-term survival in resected PDAC.

Dal Molin et al. comprehensively investigated the relationship between tumor genetic mutations and survival exceeding 10 years after resection but did not report meta-analyzable results. In a group of 35 patients who survived at least a decade after surgery, their investigation found KRAS mutations to be the most prevalent (94%) in survivors’ tumors, followed by TP53 (69%), SMAD4 (26%), CDKN2A (17%), RNF43 (11%), BRAF (5.7%), and GNAS (2.8%) mutations. However, their data showed that the long-term survival achieved by some PDAC patients cannot be attributed to differences in mutations.

**Lymphatic, Vascular, and Perineural Invasion**

Meta-analysis of data from 6 studies showed that the odds of finding lymphatic invasion were markedly lower in long-term survivors and a statistically significant odds ratio of 0.44 (95% CI: 0.32,0.60) was obtained.

Vascular invasion was found to have a negative relationship with LTS; data from 12 studies was pooled to obtain an odds ratio of 0.50 (95% CI: 0.39,0.64).

9 studies reported poolable data on the odds of finding perineural invasion in LTS patients versus NLTSs. A statistically significant overall odds ratio of OR: 0.45 (95% CI: 0.29-0.69) was computed.

**AJCC/UICC Stage**

The data on tumor staging was meta-analyzed in two different approaches.

In approach A, events were defined as tumors staged as IIB or higher. 13 studies were poolable in this manner, and an overall odds ratio of 0.36 (95% CI: 0.31 - 0.42) was obtained.

In the second approach the cut-off was set marginally higher at stage III. A slightly different set of 13 studies was meta-analyzed yielding an overall odds ratio of 0.29 (95% CI: 0.20- 0.41).

Bengtsson et al. did not report poolable data, but found on multivariate analysis that, using T1 stage as reference, LTS patients had significantly lower odds of more advanced T, N, and M stages.

**Lymph Node Metastases**

Defining an N1 status as an event, data from 21 studies were pooled to assess the odds of finding N1 in long-term survival patients. An odds ratio of 0.38 (95% CI: 0.32, 0.44) was obtained.

**Surgical and Management-Related Factors Associated with Long-term Survival**

**Adjuvant Therapy**

Some studies reporting the association between adjuvant therapy and long-term survival did not distinguish between patients receiving one of or both chemotherapy and radiation. Furthermore, many studies preceded the introduction of more contemporary chemotherapeutic agents such as gemcitabine and nab-paclitaxel. As such, 3 alternate meta-analyses were performed on data regarding the effect of adjuvant therapy.

In the first approach, events were defined as the administration of adjuvant chemotherapy and/or radiation to patients. Data were pooled from 14 studies and an overall odds ratio of 1.68 (95% CI: 1.24-2.28) was seen, suggesting an association between this management approach and increased chances of long-term survival.

In the second approach, only 10 studies where patients were specifically stated to have received chemotherapy alone were analyzed. An insignificant result was obtained, with an odds ratio of 1.56 (95% CI: 0.98-2.48).

Finally, 6 studies where adjuvant therapy included gemcitabine or nab-paclitaxel were pooled and an insignificant odds ratio of 2.28 (95% CI: 0.97 - 5.35) was obtained. However, this approach was not comprehensive as many studies did not provide specifics on the components of their chemotherapy regimens or did not specify the subgroups of patients who received gemcitabine or nab-paclitaxel. As such, this result may be discounted, or interpreted with caution at best.

**Neoadjuvant Therapy**

6 studies reported meta-analyzable data on the association between LTS and the use of neoadjuvant therapy. An insignificant overall odds ratio of 1.27 (95% CI: 0.69, 2.33) was obtained. However, these results were subgrouped due to heterogeneity and lack of comparability of the pooled studies.

Conversely, Nakano et al. only administered neoadjuvant therapy to patients with T3/T4 tumours and a significant positive association between neoadjuvant therapy and LTS was seen [OR: 5.12 (95% CI: 2.04, 12.08)]

3 studies gave neoadjuvant therapy to both resectable and borderline resectable patients. All 3 studies and the meta-analyzed result all showed no significant association between LTS and neoadjuvant therapy in these cohorts.

Paniccia et al. did not report poolable data but, using a multivariate logistic regression model; their study also found no significant association between neoadjuvant chemotherapy use and long-term survival (OR [95% CI]: 1.1[0.4-2.5]).

**Resection Margins**

Defining any outcome other than R0 resection as an event, data from 21 studies were meta-analyzed and a significant odds ratio of 0.41 (95% CI: 0.35, 0.49) was found.

**Hospital Volume**

No meta-analysis was possible to determine the association between hospital volume and the odds of patients achieving LTS. However, Huhta et al. reported a significant association between greater LTS and higher quintiles of annual hospital volume (HR 1.43 [1.16–1.75]).

**Operative Blood Loss**

4 studies’ data were meta-analyzed and long-term survivors were found to have significantly less operative blood loss than other patients. A mean difference of –545.95ml (95% CI: -804.2, -287.39) was obtained.

Picozzi et al dichotomized operative blood loss at 250ml, precluding pooling of their data. They did not find a significant difference between LTSs and NLTSs.

**Operative Time**

Meta-analysis of 4 studies showed a mean difference of -33.88 minutes (95% CI: -59.60, -8.16), with shorter surgeries significantly associated with long-term survival.

**Intraoperative Radiotherapy**

Two studies were pooled to show no significant association between the use of intra-operative radiation therapy and the attainment of long-term survival. An odds ratio of 1.20 (95% CI: 0.65, 2.22) was computed.

**Perioperative Blood Transfusion**

Only 1 of 4 pooled studies independently showed a significant association between decreased odds of blood transfusion perioperatively in patients who survived long-term versus those who did not. However, the point estimates for all 4 studies supported such an association, and meta-analysis showed a significant result overall, with an odds ratio of 0.52 (95% CI: 0.35, 0.76)

**Vascular Resection**

Data from 7 articles, of which 6 reported null findings, were meta-analyzed as a composite outcome that defined the resection of blood vessels as an event. A significant association with long-term survival was seen, with an odds ratio of 0.62 (95% CI: 0.41-0.93).

2 studies were sub-grouped under portal venous resection only, with an odds ratio of 0.79 (95% CI: 0.36, 1.72). 2 studies were sub-grouped as portal venous and/or superior mesenteric venous resection, with an odds ratio of OR: 0.51 (95% CI: 0.11, 2.38). The 3 remaining studies were sub-grouped with events defined as any vascular resection, and an odds ratio of 0.62 (95% CI: 0.41 - 0.93) was computed.

**Hospital Stay**

Duration of hospital stay was not found to be significantly associated with long-term survival. Using data pooled from 3 studies, an overall mean difference of –0.77 days (95% CI: -3.17, 1.61) was found.

Picozzi et al. analyzed length of stay as a dichotomous variable greater than or less than 10 days and found no significant association with achievement of LTS.

**Major Post-operative Morbidity and Complications**

Events were defined as Clavien-Dindo categorization of greater than or equal to 3. Data were pooled from 3 studies and no significant association was found with long-term survival – an odds ratio of 0.81 (95% CI: 0.47-1.39) was calculated.

Nakano et al. and Dusch et al. reported morbidity data in a non-poolable manner (due to the possibility of double counting patients). However, both studies also obtained null results for the association between LTS and post-operative complications/morbidity.

**NCI Designated Cancer Centre**

No meta-analysis was possible for this outcome. However, Kardosh et al. reported a significant association (OR [95% CI]: 1.34 [1.12–1.61]) between increased odds of LTS and treatment at an NCI cancer center.

**Section 6: Forest Plots**

**Legend:** M-H, Mantel-Haenszel; SD, standard deviation; IV, inverse variance; CI, confidence interval; df, degrees of freedom; P, probability value

**Meta-Analyses of Continuous Data (Mean Differences):**

**Supplementary Figure 5.1: Age (in Years)**

**
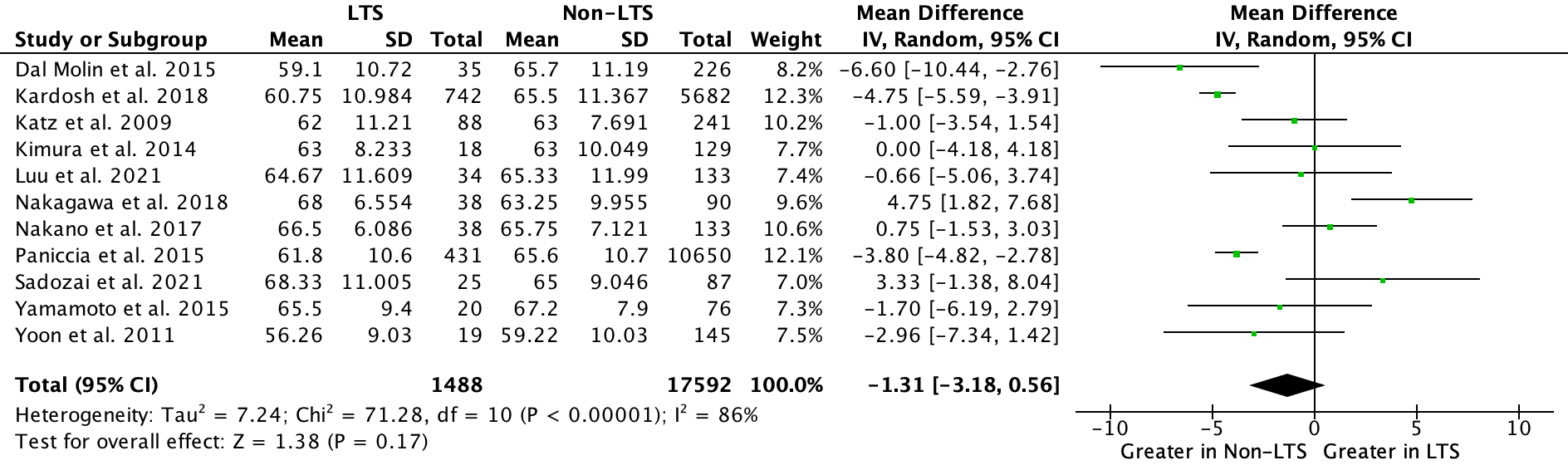
**

**Supplementary Figure 5.2: BMI (kg/m^2^)**

**
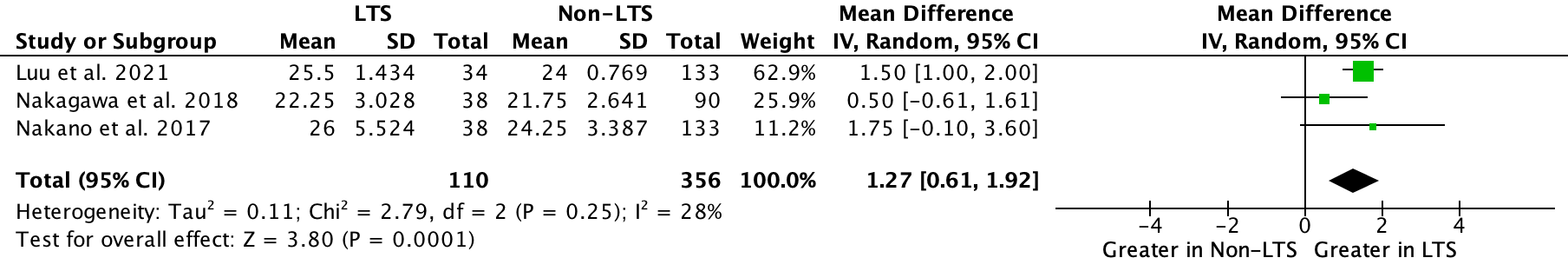
**

**Supplementary Figure 5.3: Preoperative CEA (ng/mL)**

**
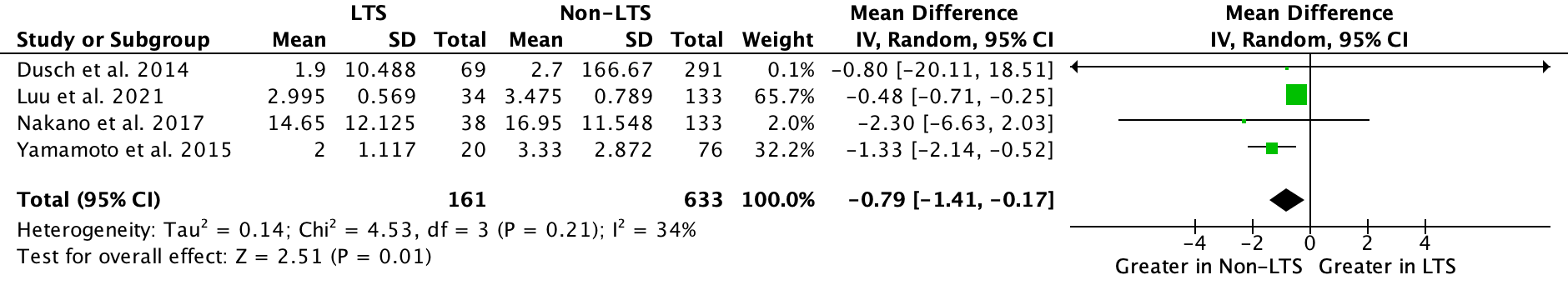
**

**Supplementary Figure 5.4: Preoperative CA19-9 (units/mL)**

**
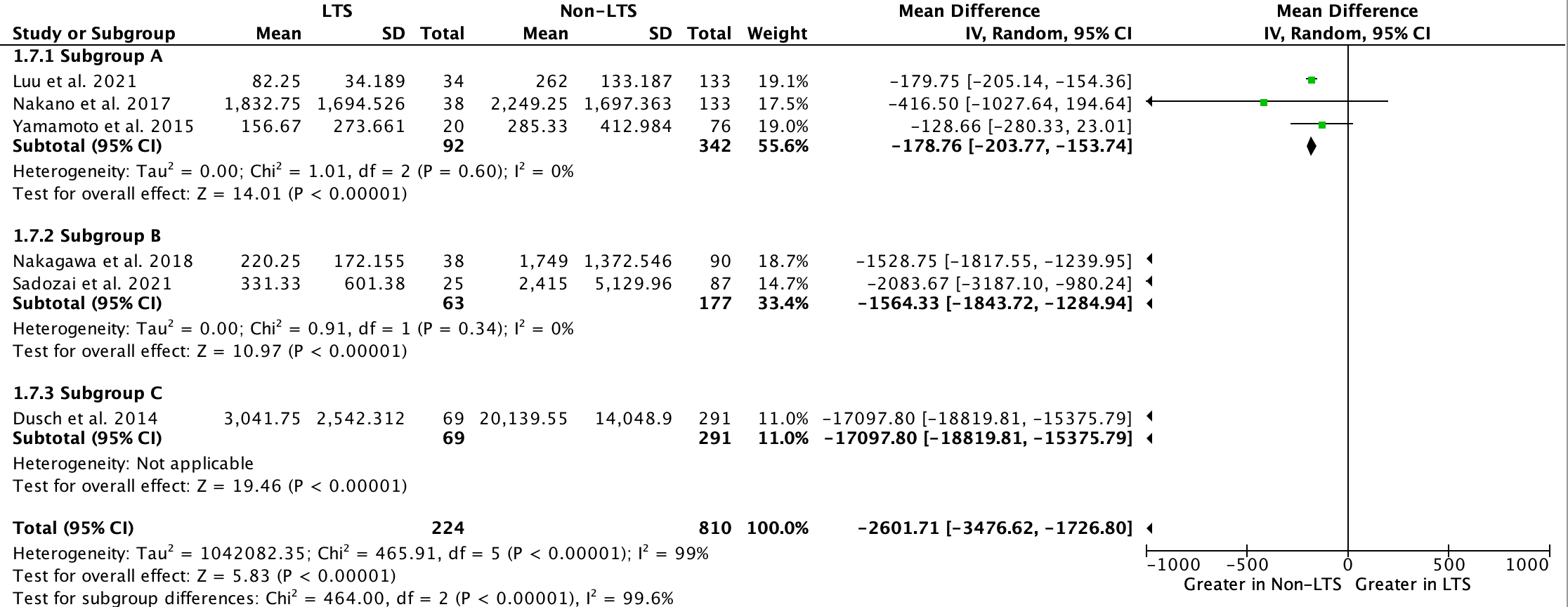
**

**Supplementary Figure 5.5: Preoperative Albumin (mg/mL)**

**
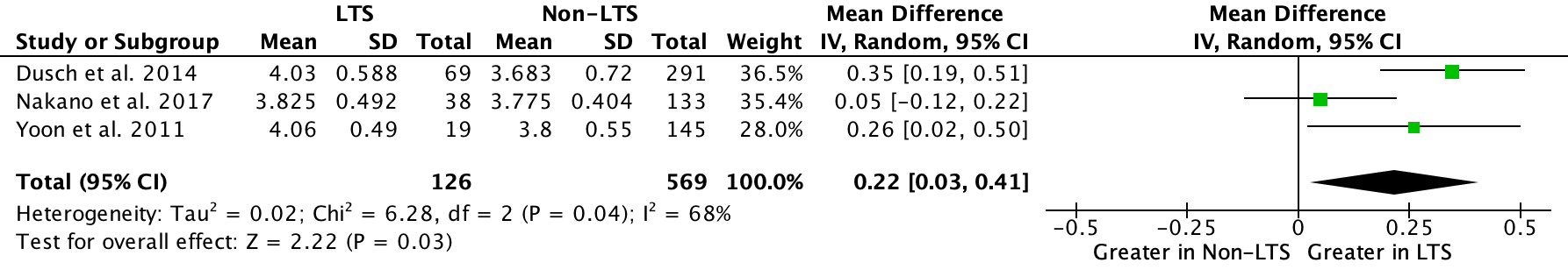
**

**Supplementary Figure 5.6: Preoperative Bilirubin (mg/dL)**

**
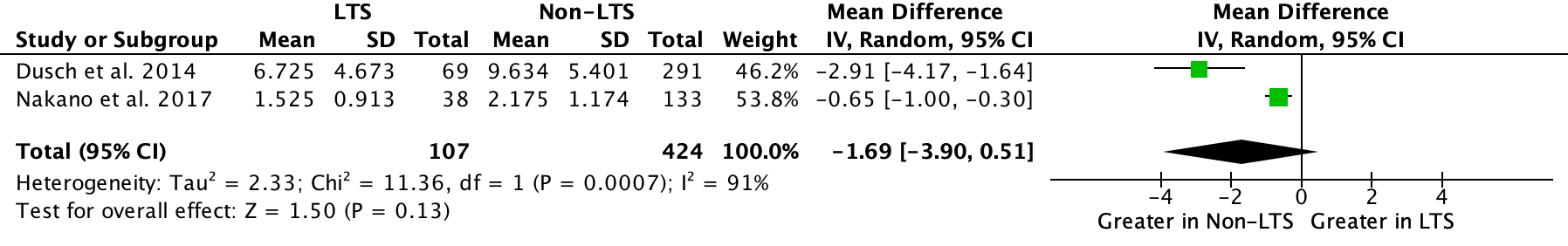
**

**Supplementary Figure 5.7: Tumor Size (cm)**

**
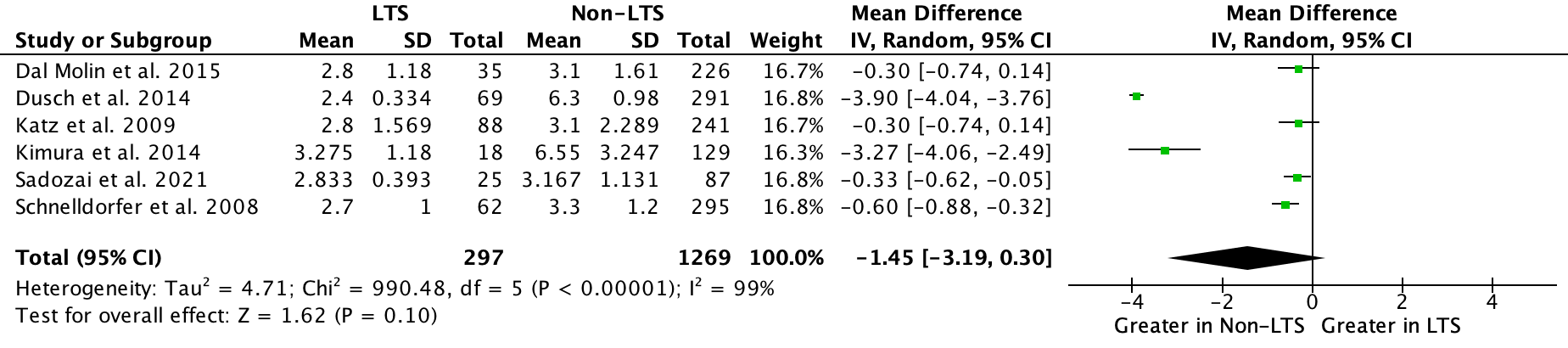
**

**Supplementary Figure 5.8: Operative Blood Loss (mL)**

**
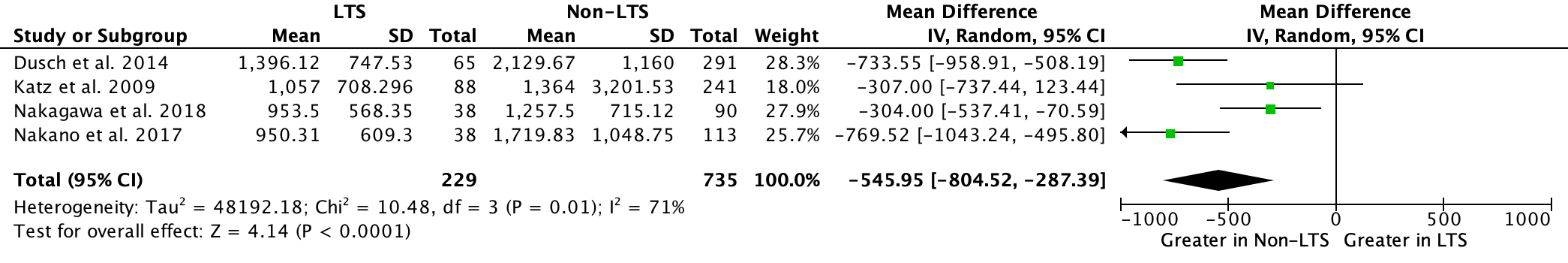
**

**Supplementary Figure 5.9: Operative Time (Minutes)**

**
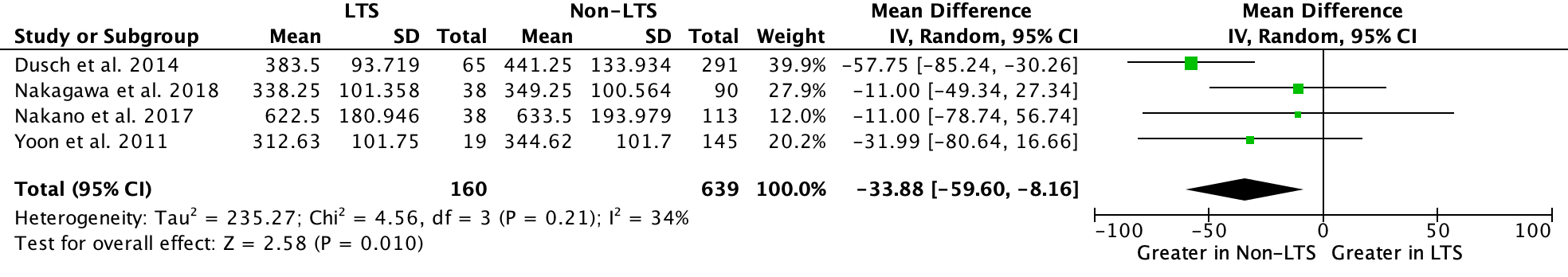
**

**Supplementary Figure 5.10: Hospital Stay (Days)**

**
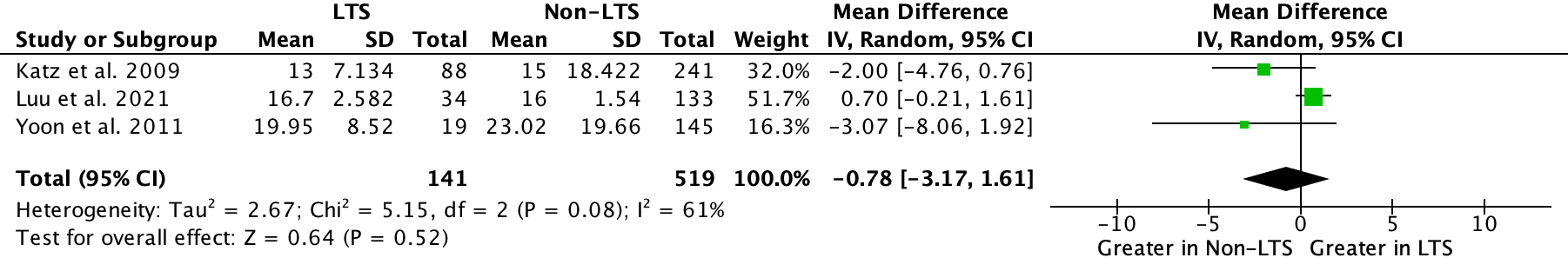
**

**Meta-Analyses of Categorical Data (Odds Ratios):**

**Supplementary Figure 5.11: Female Sex**

**
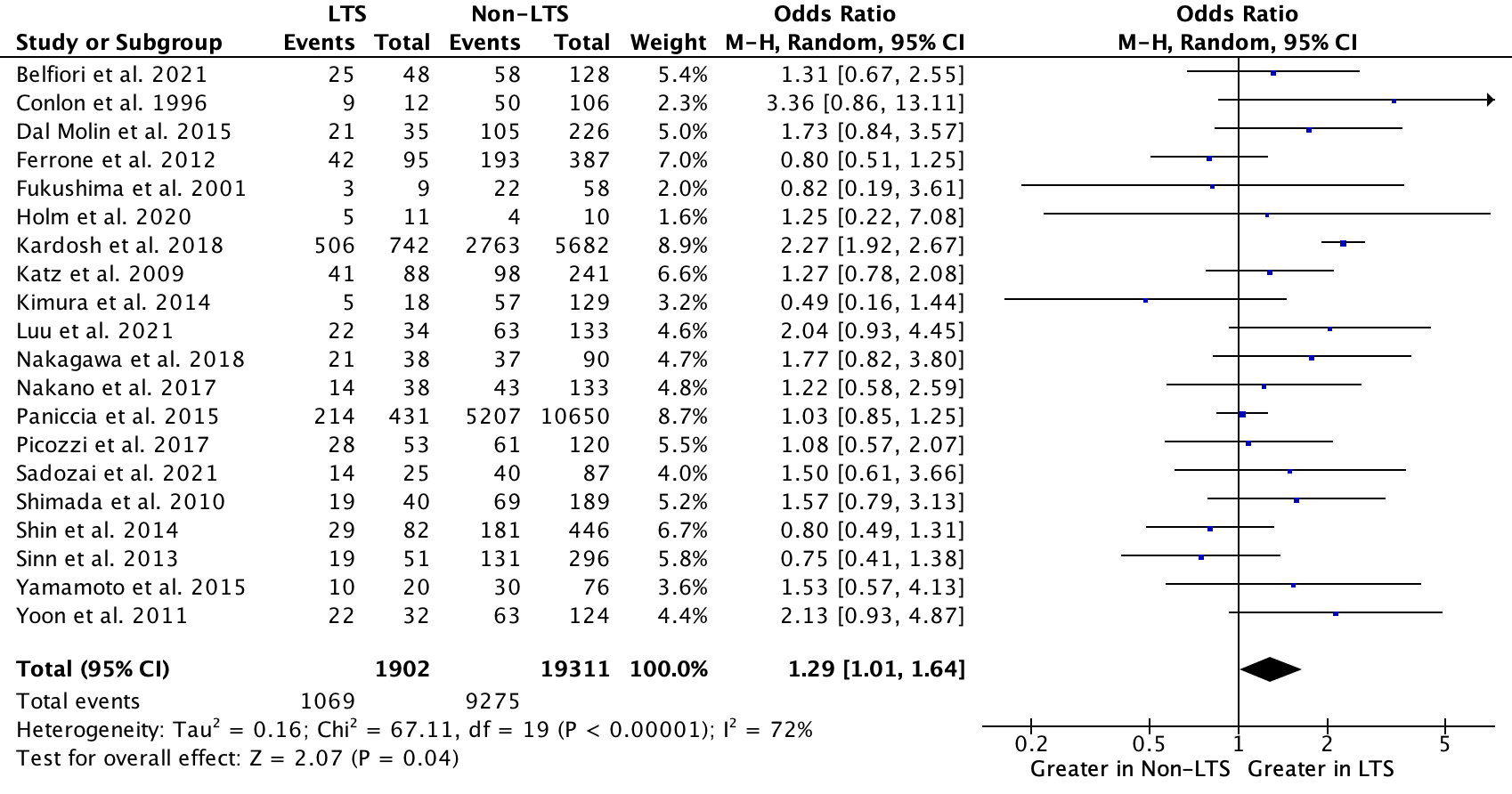
**

**Supplementary Figure 5.12: Preoperative Biliary Stenting**

**
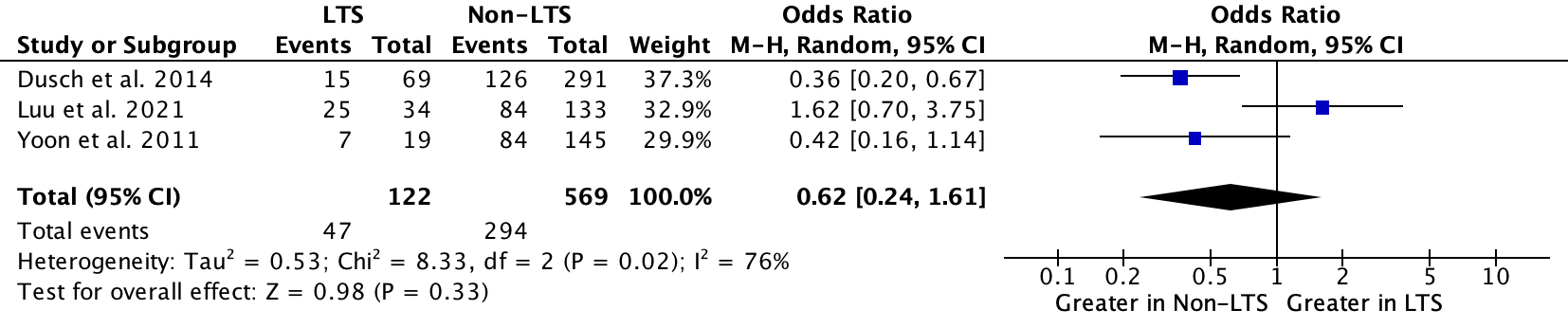
**

**Supplementary Figure 5.13: Diabetes**

**
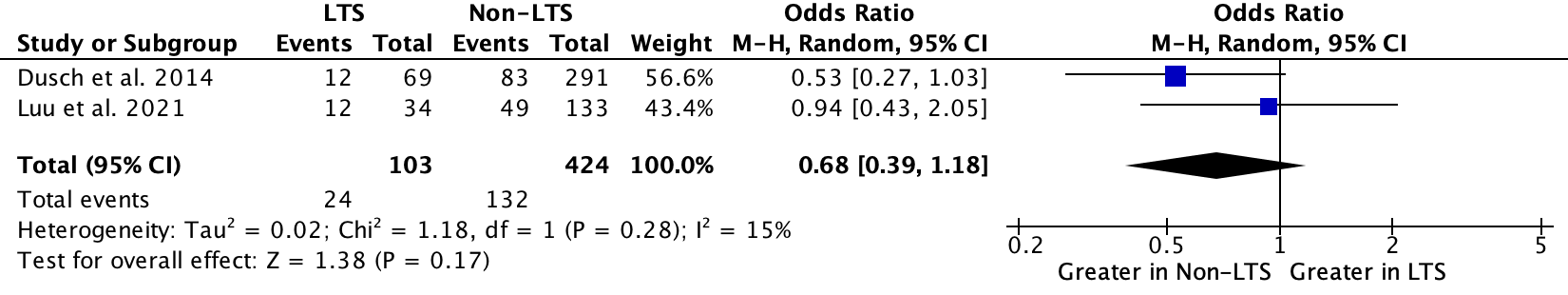
**

**Supplementary Figure 5.14: Preoperative ASA Classification (ASA≥3)**

**
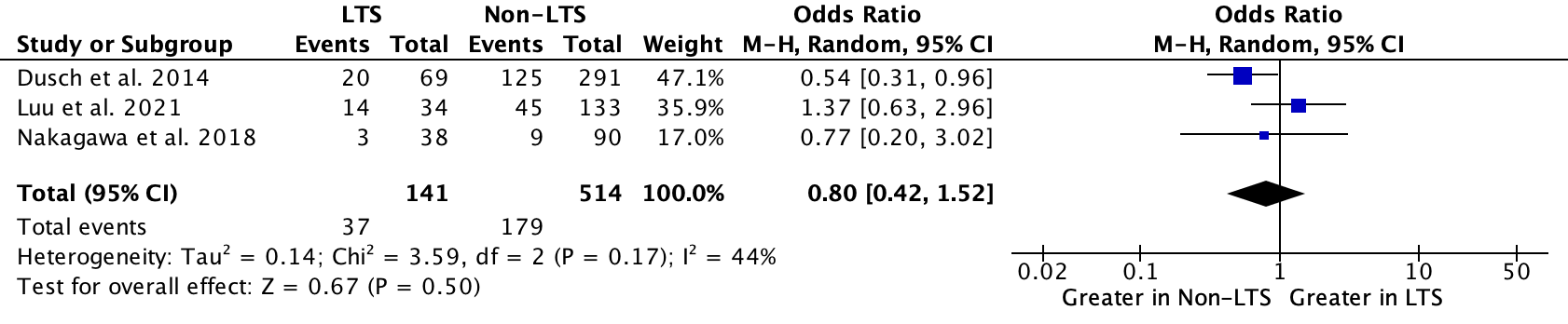
**

**Supplementary Figure 5.15: Alcohol Use**

**
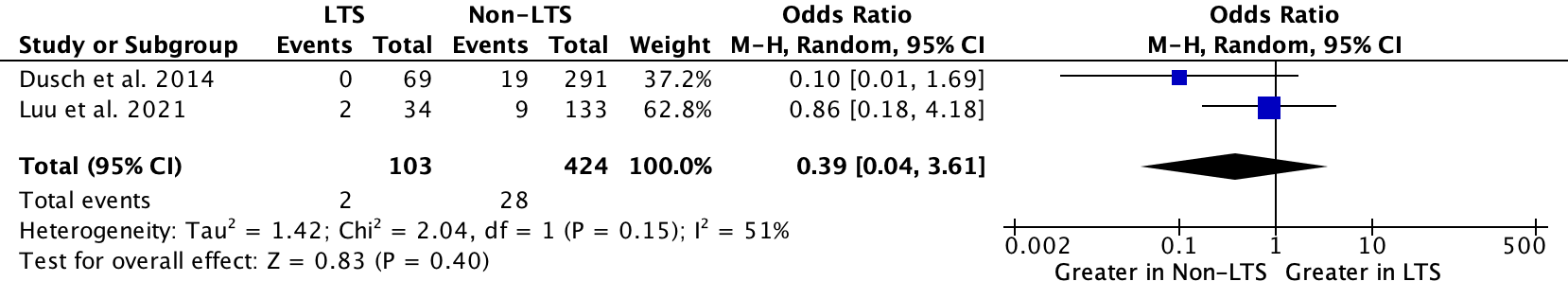
**

**Supplementary Figure 5.16: Cardiovascular Disease**

**
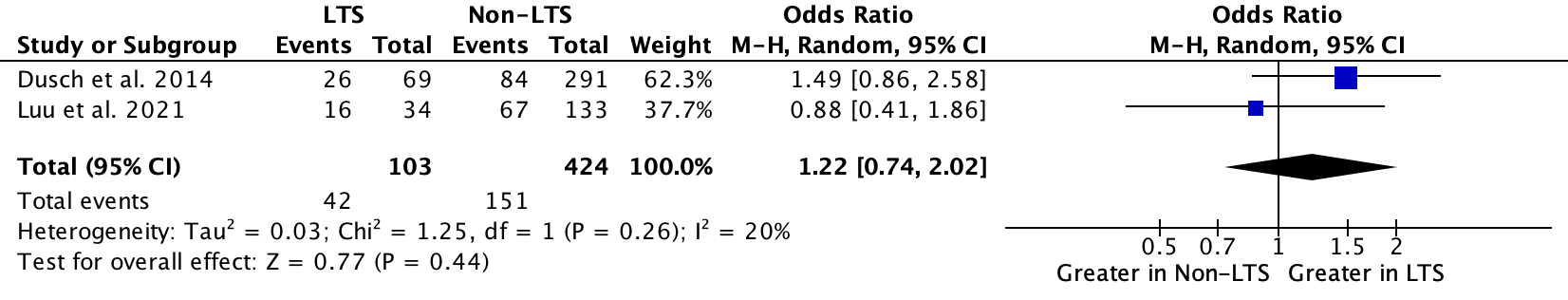
**

**Supplementary Figure 5.17: Lymph Node Metastasis**

**
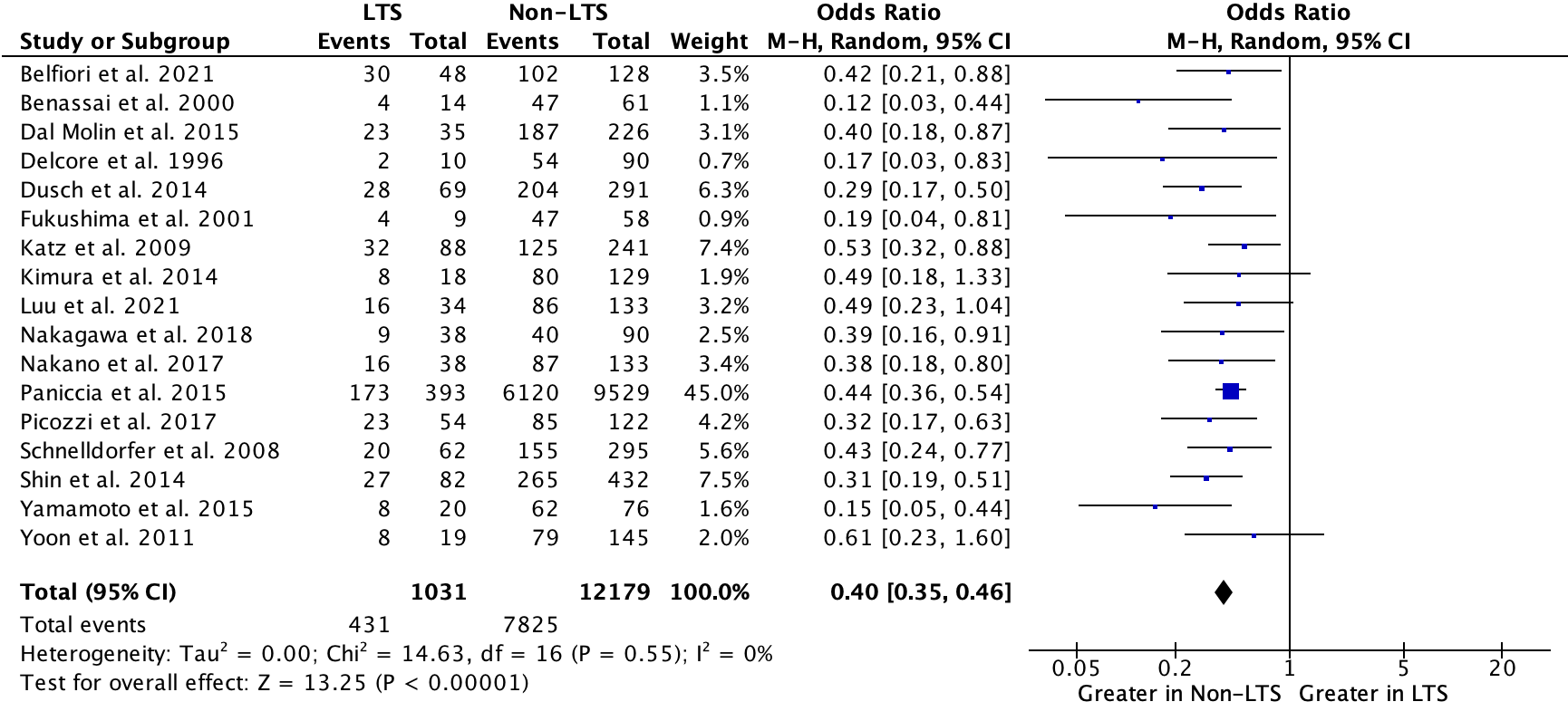
**

**Supplementary Figure 5.18: Tumour Grade (≥Grade III vs. <Grade III)**

**
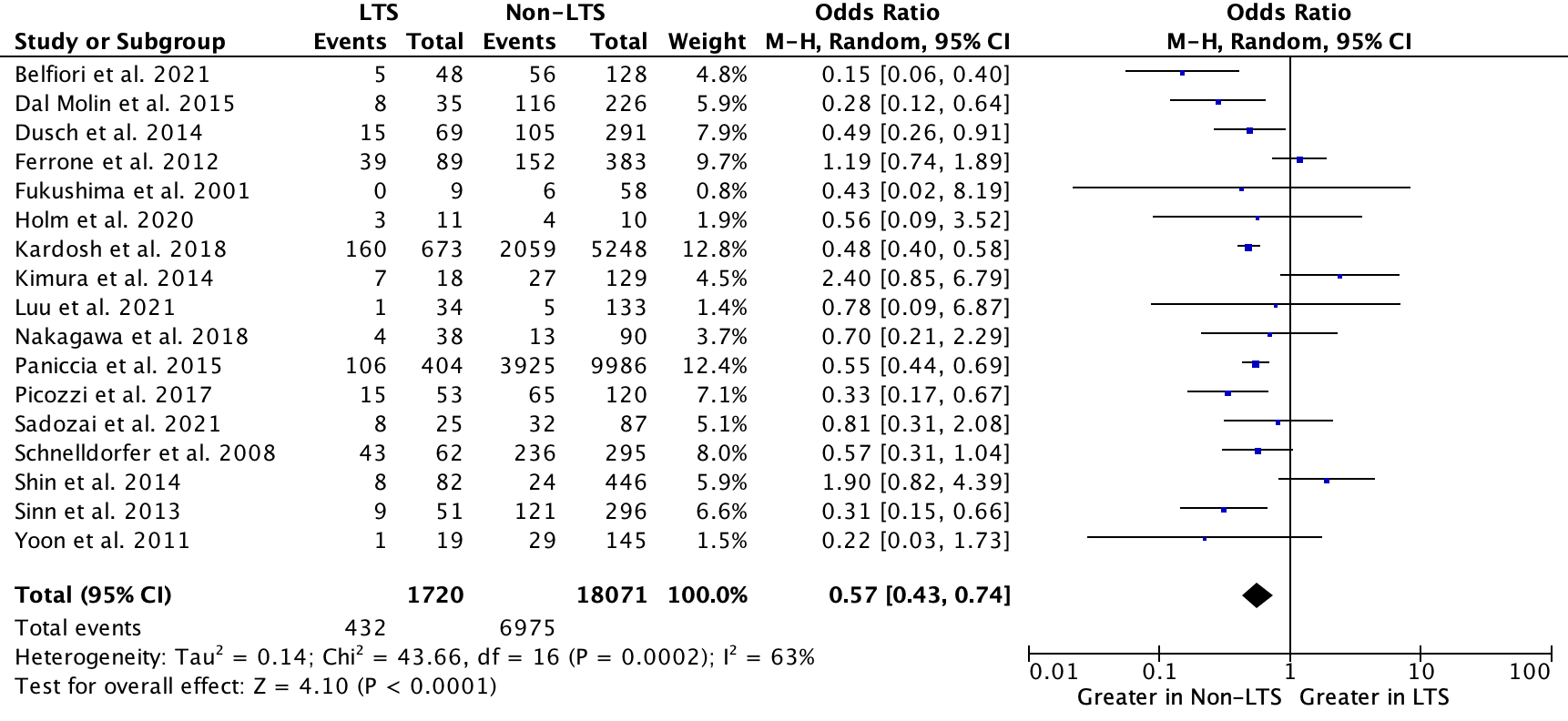
**

**Supplementary Figure 5.19: AJCC/UICC Stage (≥Stage IIB vs. <Stage IIB)**

**
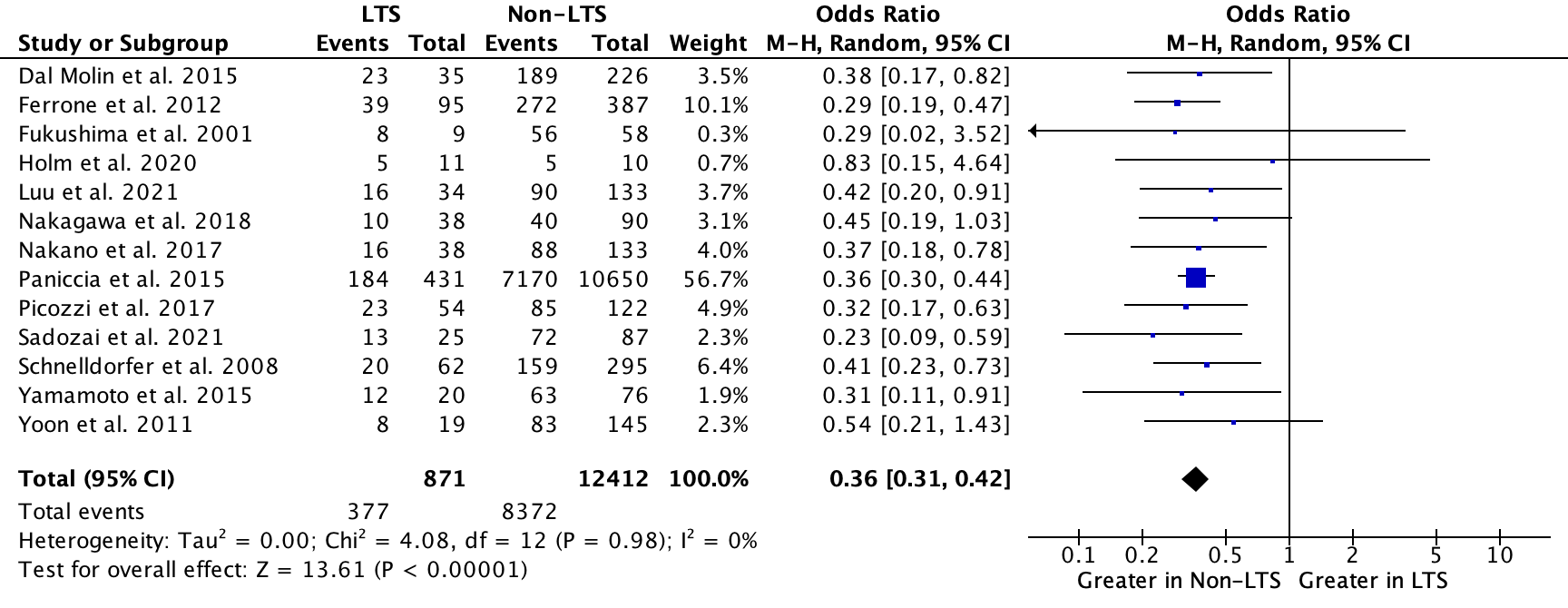
**

**Supplementary Figure 5.20: AJCC/UICC Stage (≥Stage III vs. <Stage III)**

**
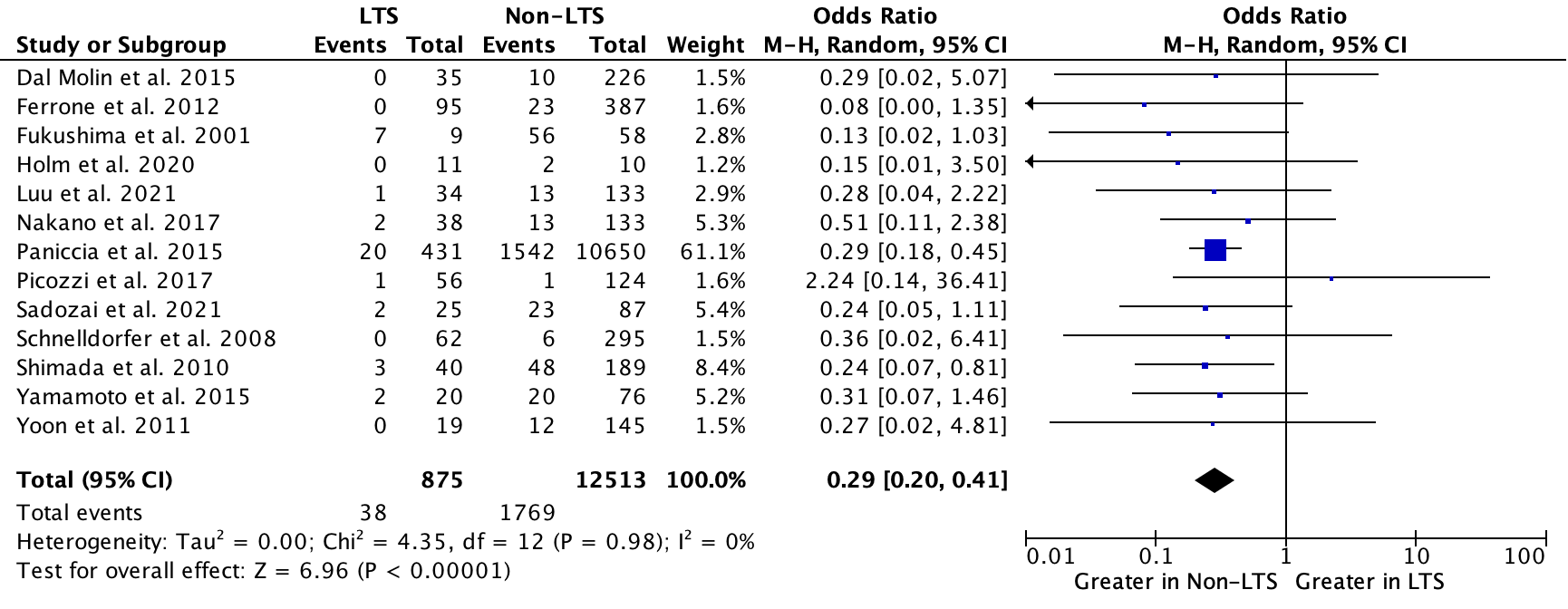
**

**Supplementary Figure 5.21: Pathologic T-stage (≥T3 vs. <T3)**

**
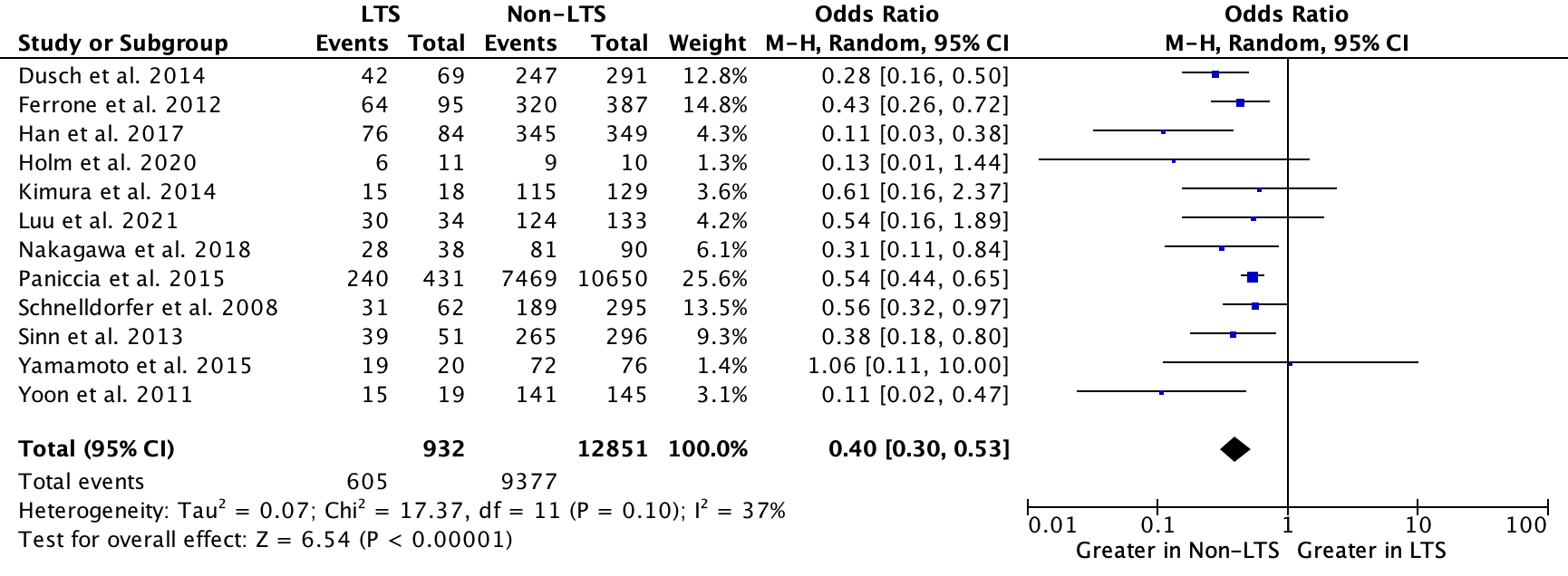
**

**Supplementary Figure 5.22: Tumour Location (Pancreatic Head vs. Other Locations)**

**
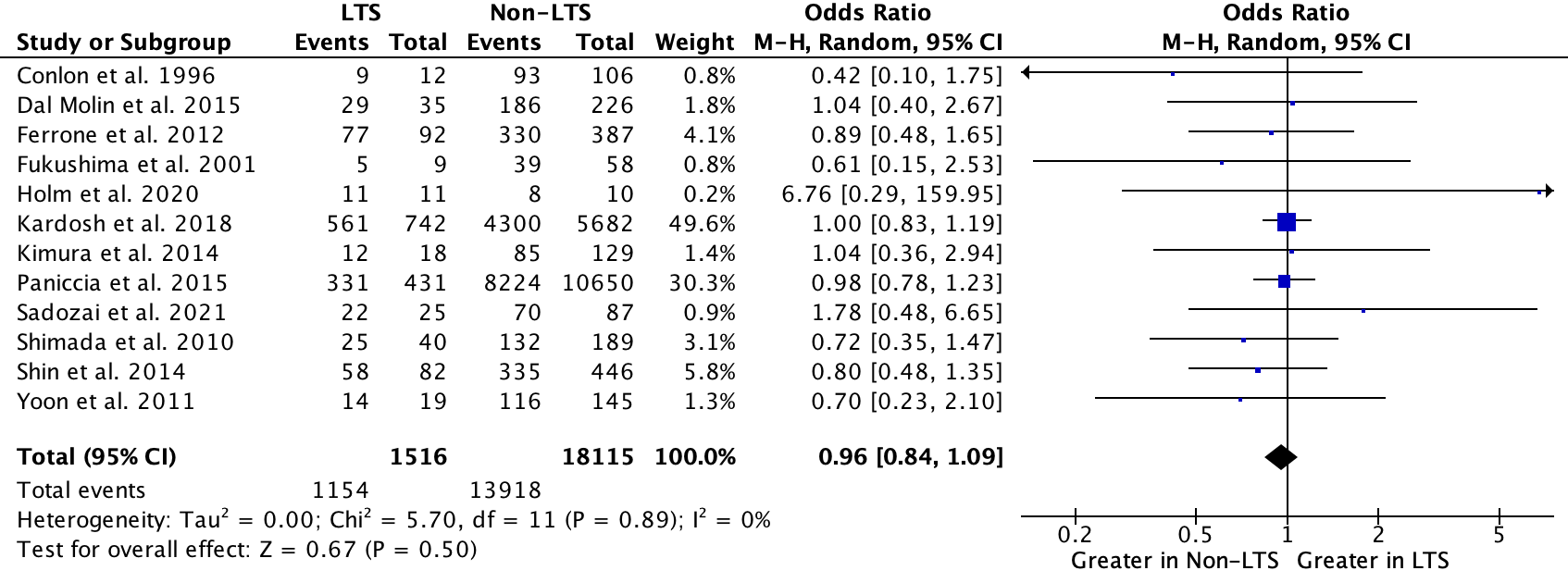
**

**Supplementary Figure 5.23: Vascular Invasion**

**
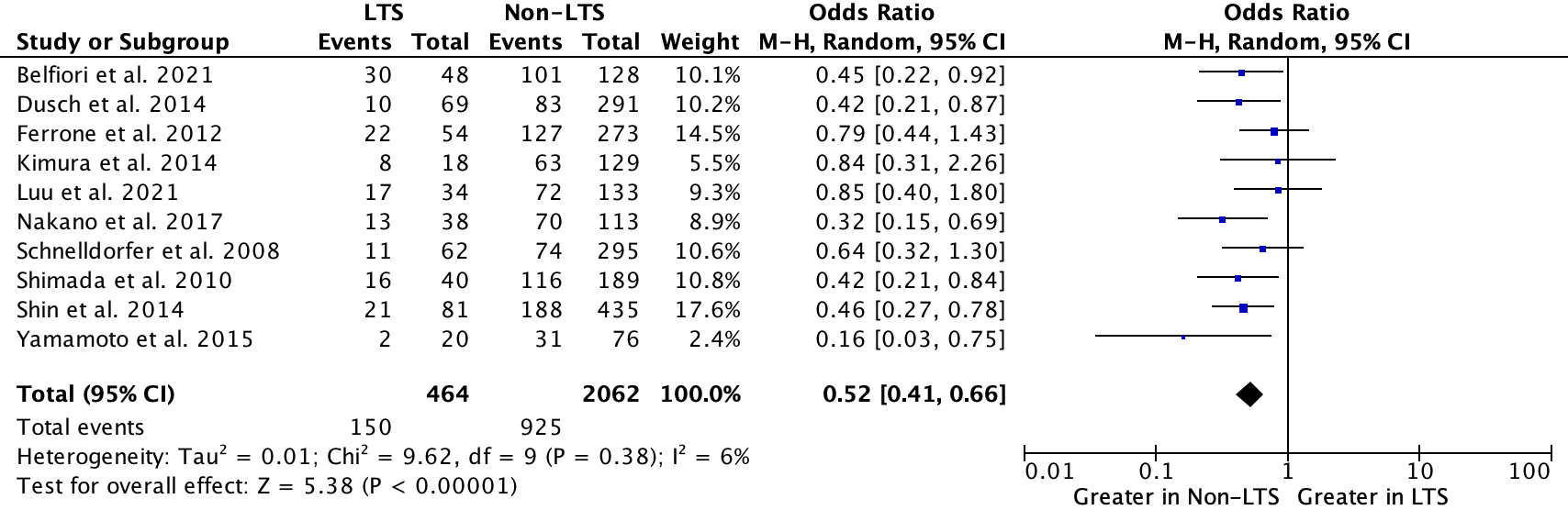
**

**Supplementary Figure 5.24: Perineural Invasion**

**
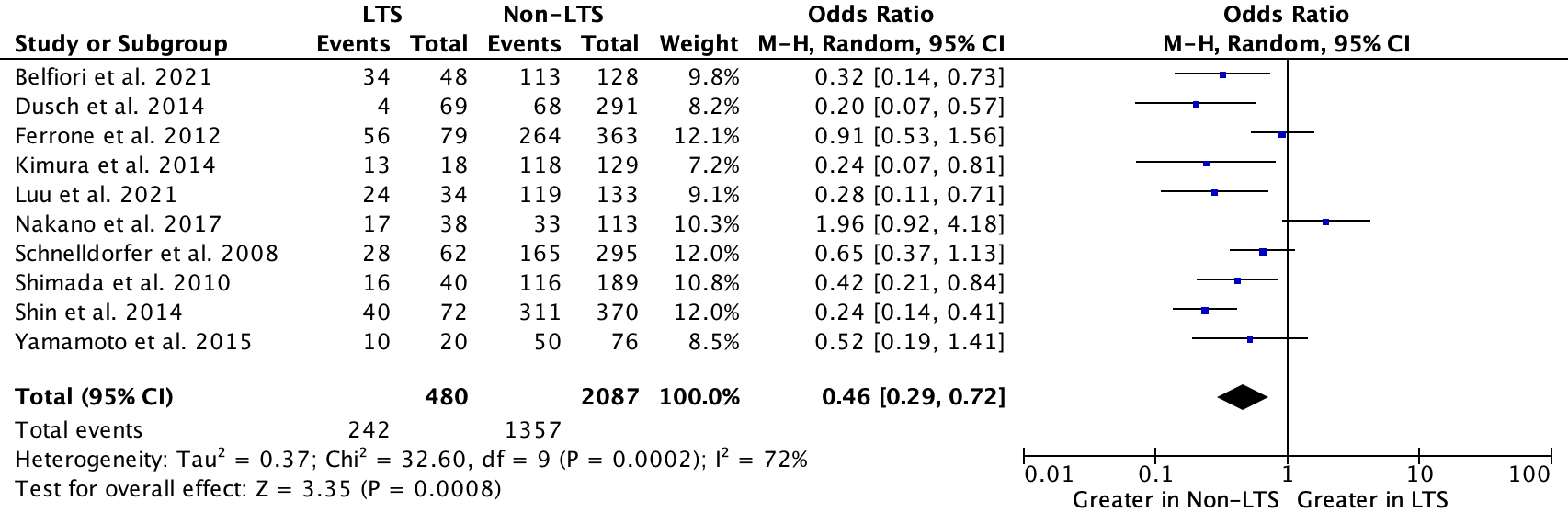
**

**Supplementary Figure 5.25: Tumour Grade (≥Grade II vs. Grade I)**

**
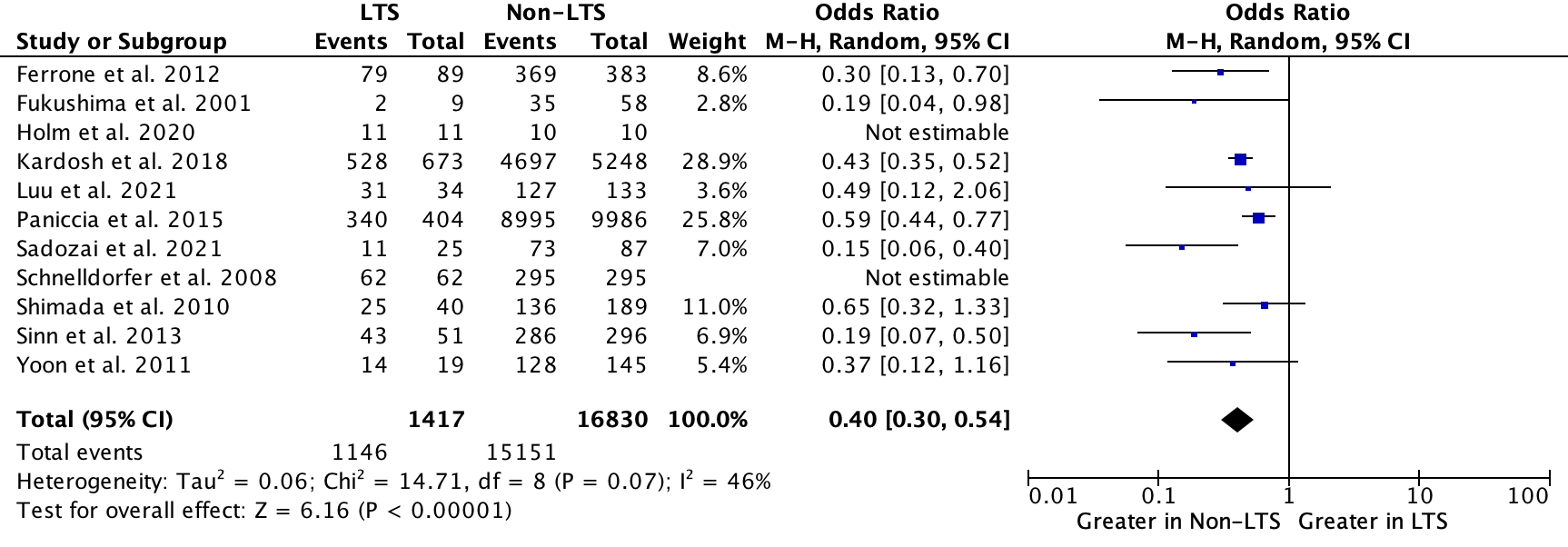
**

**Supplementary Figure 5.26: Lymphatic Invasion**

**
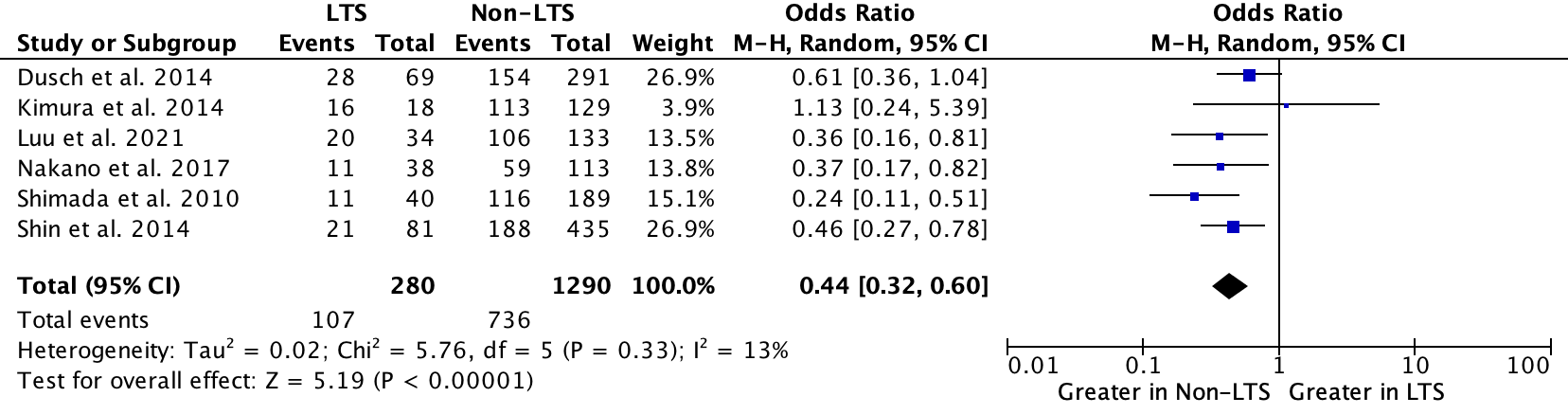
**

**Supplementary Figure 5.27: Pathologic M-Stage**

**
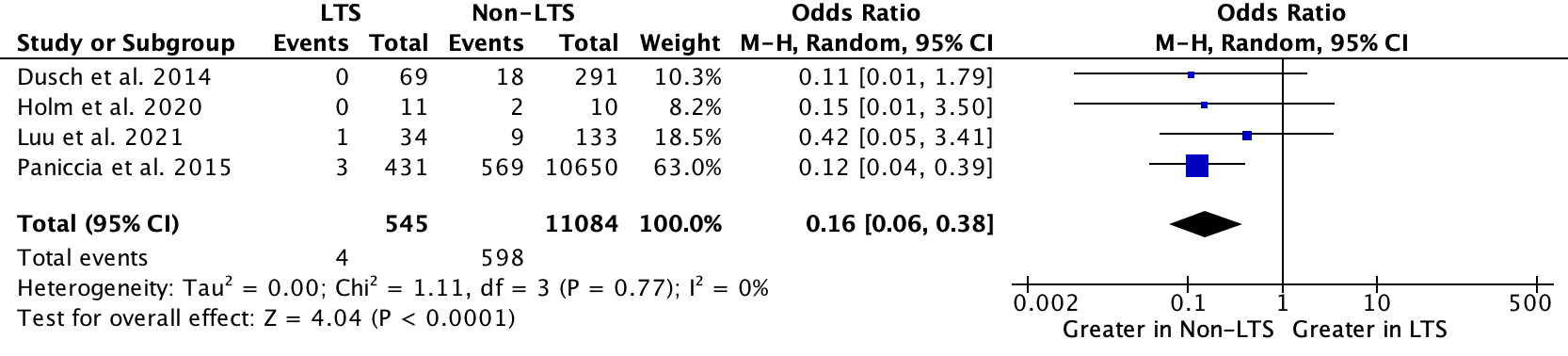
**

**Supplementary Figure 5.28: Positive Resection Margins**

**
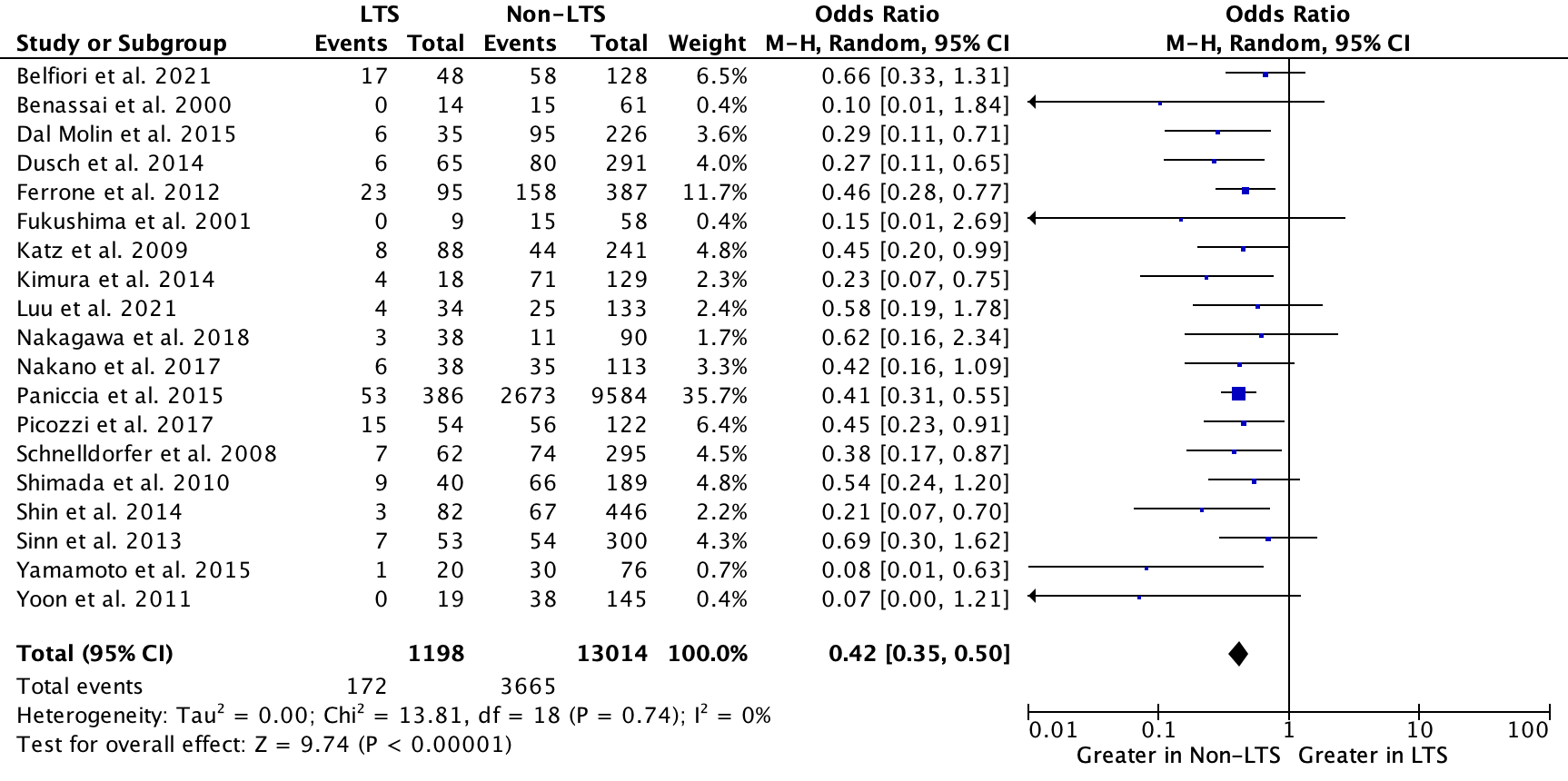
**

**Supplementary Figure 5.29: Adjuvant Therapy (Chemotherapy and/or Radiotherapy)**

**
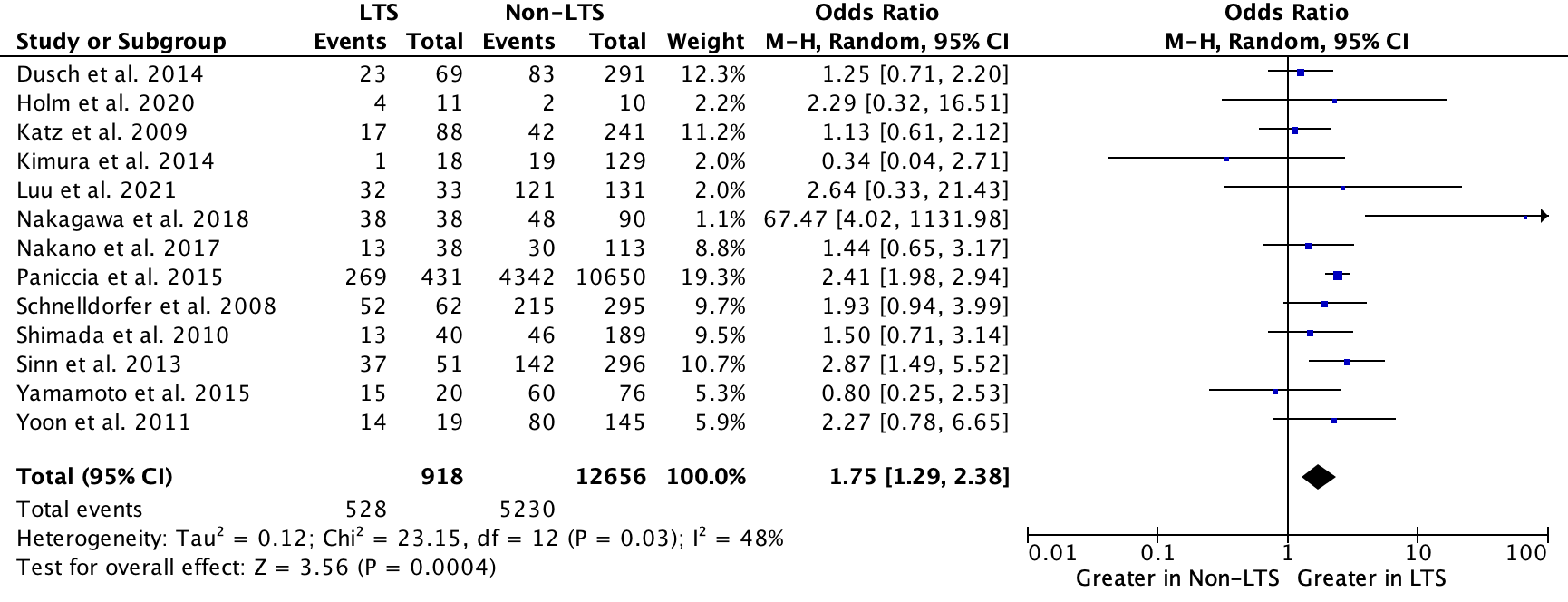
**

**Supplementary Figure 5.30: Adjuvant Therapy (Chemotherapy Only)**

**
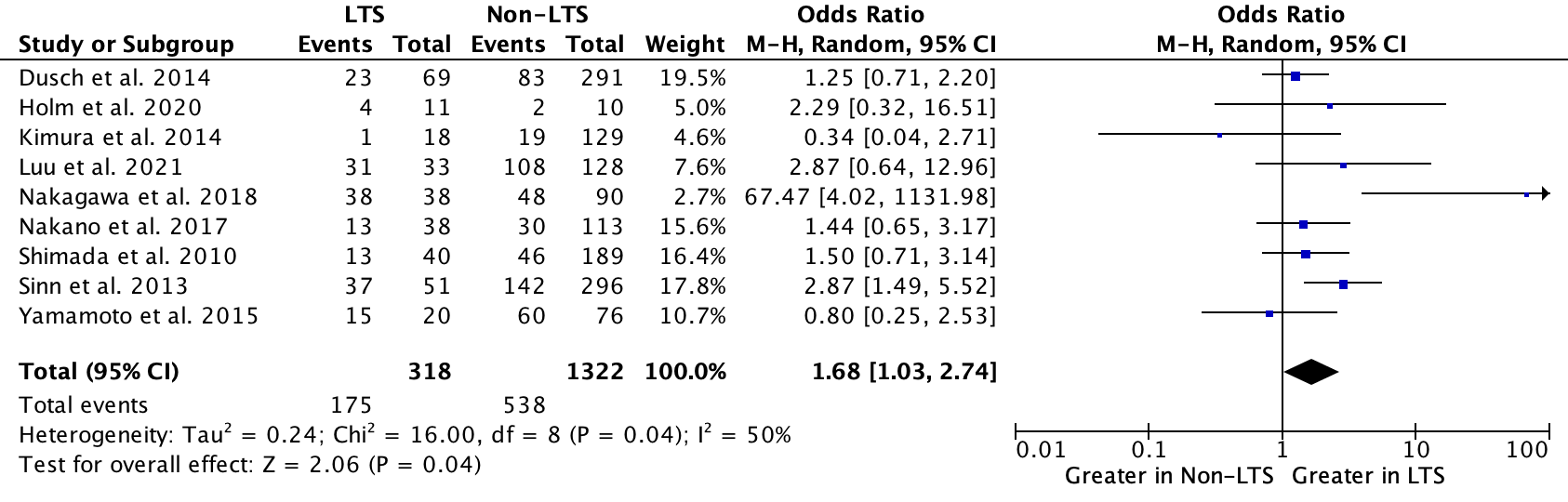
**

**Supplementary Figure 5.31: Vascular Resection**

**
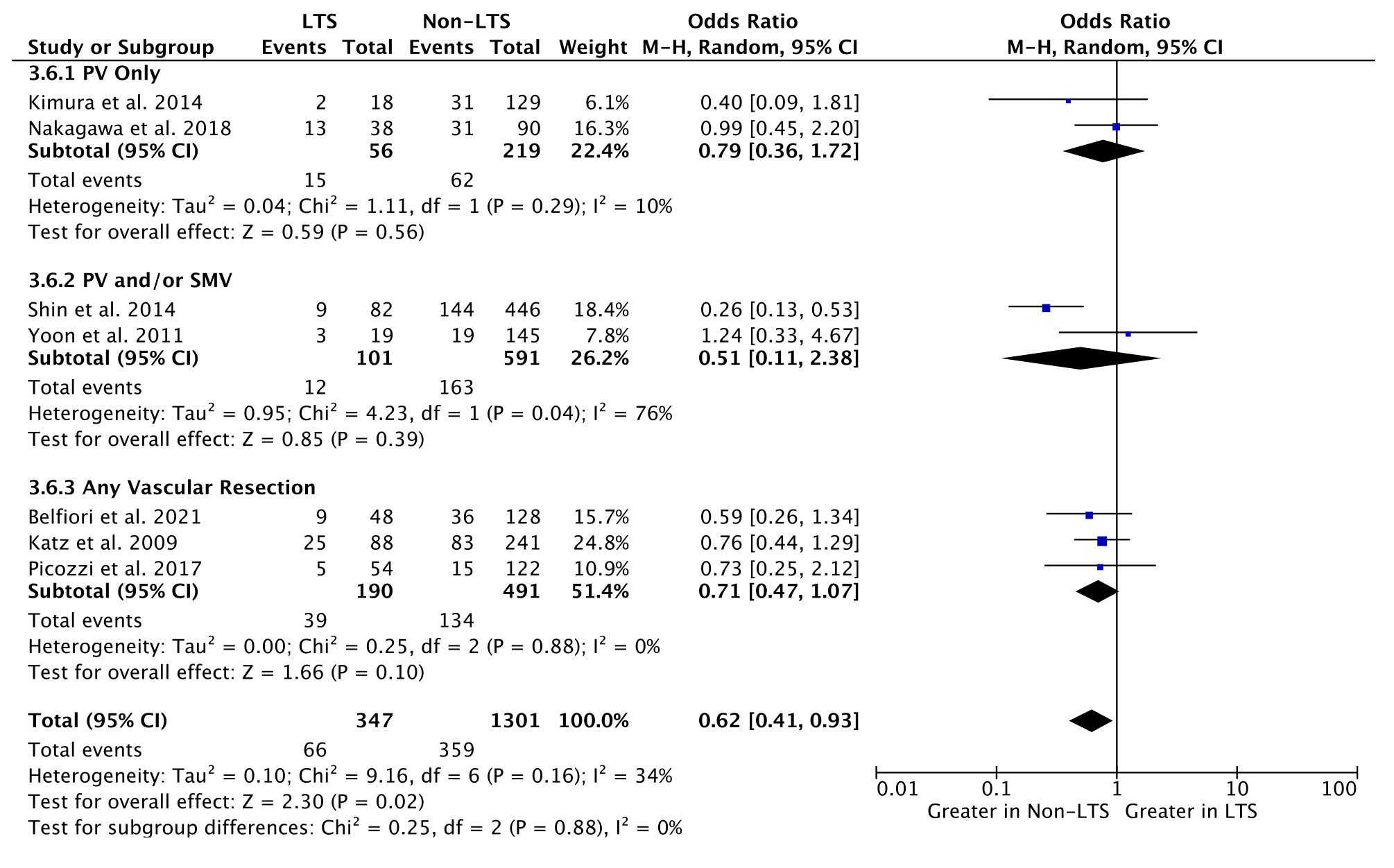
**

**Supplementary Figure 5.32: Adjuvant Therapy (Any adjuvant therapy including gemcitabine or nab-paclitaxel)**

**
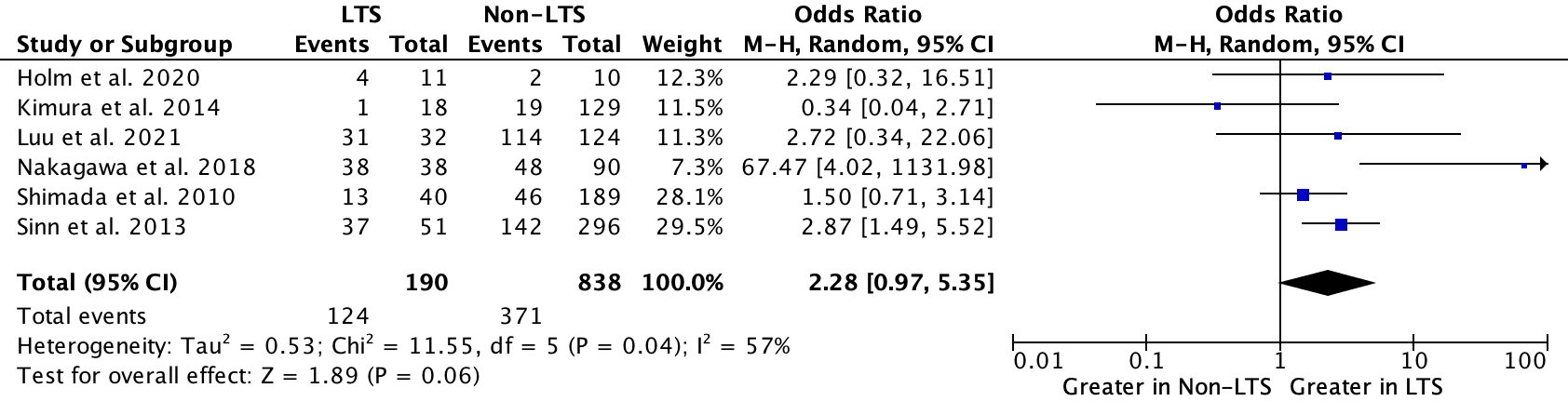
**

**Supplementary Figure 5.33: Neoadjuvant Therapy**

**
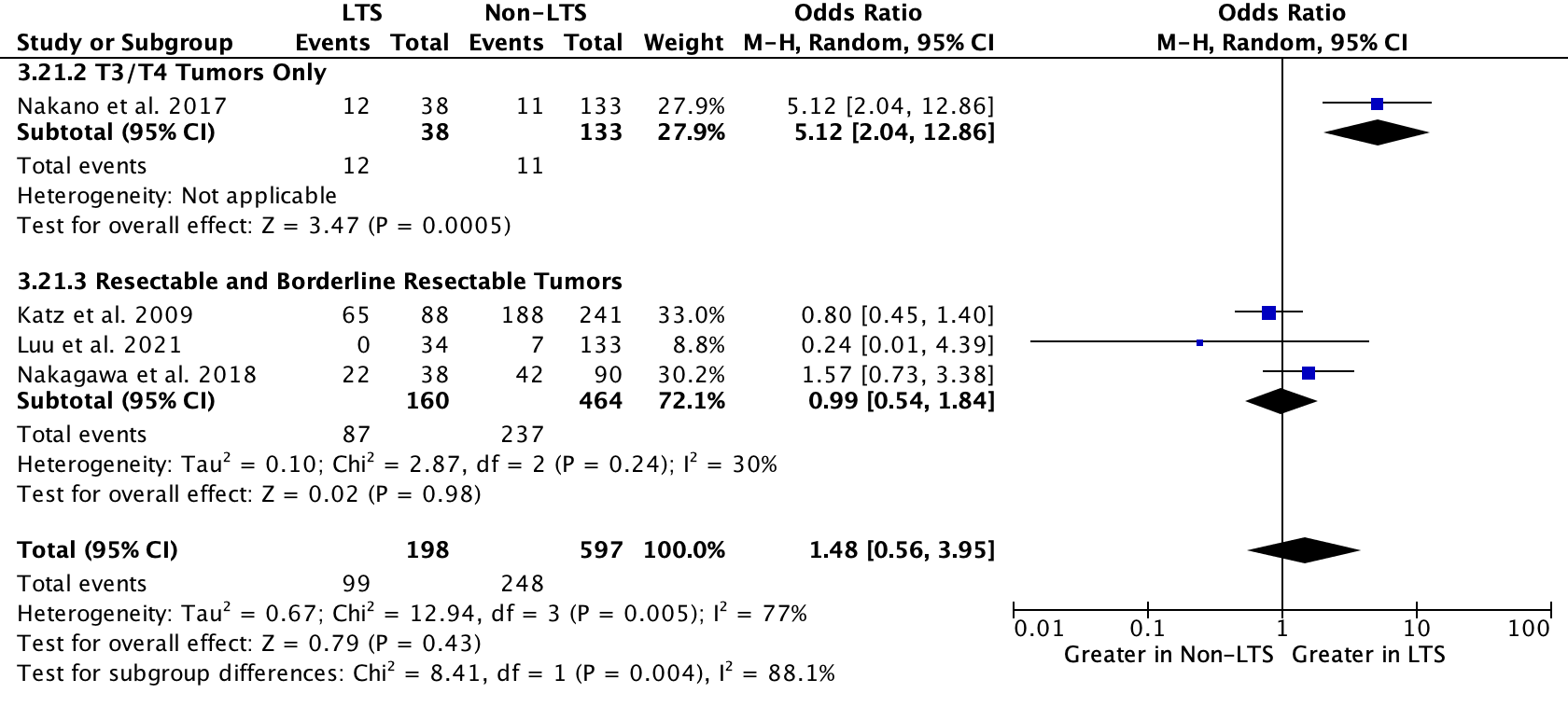
**

**Supplementary Figure 5.34: Perioperative Blood Transfusion**

**
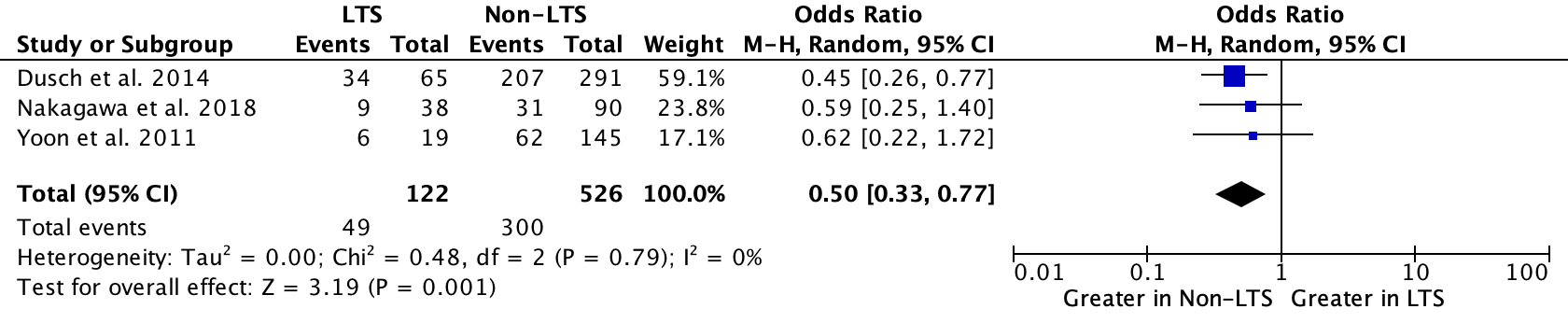
**

**Supplementary Figure 5.35: Major Postoperative Morbidity/Complications**

**
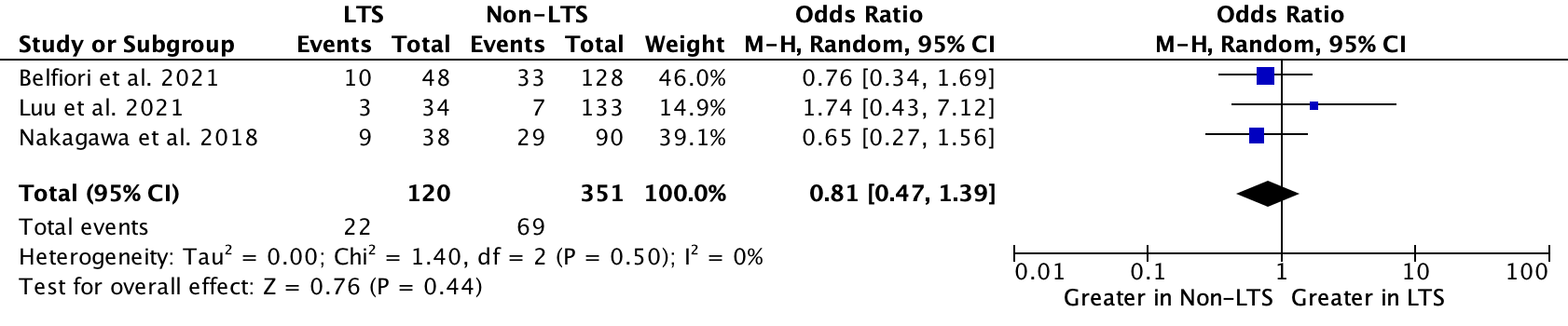
**

**Supplementary Figure 5.36: Intraoperative Radiotherapy**

**
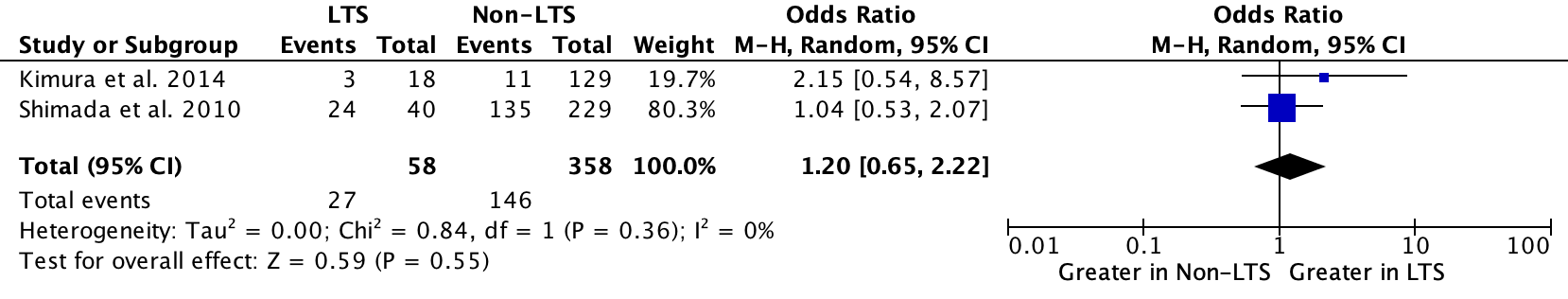
**

**Section 7: GRADE Evidence Profile**

**Patient Characteristics**

| **Certainty assessment** | | | | | | | **№ of patients** | | **Effect** | | **Certainty** | **Importance** |
| --- | --- | --- | --- | --- | --- | --- | --- | --- | --- | --- | --- | --- |
| **№ of studies** | **Study design** | **Risk of bias** | **Inconsistency** | **Indirectness** | **Imprecision** | **Other considerations** | **Long-Term Survivors** | **Non-Long-Term Survivors** | **Relative (95% CI)** | **Absolute (95% CI)** |  |  |
| **Age (years)** | | | | | | | | | | | | |
| 11 | observational studies | not serious | very serious^a^ | not serious | serious^b^ | none | 1488 | 17592 | - | MD **1.31 Years lower** (3.18 lower to 0.56 lower) | ⨁◯◯◯ Very low |  |
| **Body Mass Index (kg/m^2)** | | | | | | | | | | | | |
| 3 | observational studies | not serious | not serious | not serious | not serious | none | 110 | 356 | - | MD **1.27 higher** (0.61 higher to 1.92 higher) | ⨁⨁◯◯ Low |  |
| **Female Sex** | | | | | | | | | | | | |
| 20 | observational studies | not serious | serious^c^ | not serious | not serious | none | 1069/1902 (56.2%) | 9275/19311 (48.0%) | **OR 1.29** (1.01 to 1.64) | **64 more per 1,000** (from 2 more to 122 more) | ⨁◯◯◯ Very low |  |
| **Preoperative Bilirubin (mg/dL)** | | | | | | | | | | | | |
| 2 | observational studies | not serious | very serious^a^ | not serious | not serious | none | 107 | 424 | - | MD **1.69 lower** (3.9 lower to 0.51 higher) | ⨁◯◯◯ Very low |  |
| **Preoperative Albumin (mg/mL)** | | | | | | | | | | | | |
| 3 | observational studies | not serious | serious^c^ | not serious | not serious | none | 126 | 569 | - | MD **0.22 higher** (0.03 higher to 0.41 higher) | ⨁◯◯◯ Very low |  |
| **Diabetes** | | | | | | | | | | | | |
| 2 | observational studies | not serious | not serious | not serious | not serious |  | 24/103 (23.3%) | 132/424 (31.3%) | **OR 0.68** (0.39 to 1.18) | **76 fewer per 1,000** (from 161 fewer to 37 more) | ⨁⨁◯◯ Low |  |
| **Cardiovascular Disease** | | | | | | | | | | | | |
| 2 | observational studies | not serious | not serious | not serious | not serious | none | 26/69 (37.7%) | 84/291 (28.9%) | **OR 1.49** (0.86 to 2.58) | **88 more per 1,000** (from 30 fewer to 223 more) | ⨁⨁◯◯ Low |  |
| **Preoperative CA19-9 (units/mL)** | | | | | | | | | | | | |
| 6 | observational studies | not serious | very serious^a^ | not serious | serious^b^ | very strong association | 224 | 810 | - | MD **2601.71 lower** (3476.62 lower to 1726.8 lower) | ⨁◯◯◯ Very low |  |
| **Preoperative CA19-9 - Subgroup A** | | | | | | | | | | | | |
| 3 | observational studies | not serious | not serious | not serious | not serious | none | 92 | 342 | - | MD **178.76 lower** (203.77 lower to 153.74 lower) | ⨁⨁◯◯ Low |  |
| **Preoperative CA19-9 - Subgroup B** | | | | | | | | | | | | |
| 2 | observational studies | not serious | not serious | not serious | not serious | strong association | 63 | 177 | - | MD **1564.33 lower** (1843.72 lower to 1284.94 lower) | ⨁⨁⨁◯ Moderate |  |
| **Preoperative CA19-9 - Subgroup C** | | | | | | | | | | | | |
| 1 | observational studies | not serious | not serious | not serious | not serious | very strong association | 69 | 291 | - | MD **17097.8 lower** (18819.81 lower to 15375.79 lower) | ⨁⨁⨁⨁ High |  |
| **Preoperative CEA (ng/mL)** | | | | | | | | | | | | |
| 4 | observational studies | not serious | not serious | not serious | not serious | none | 161 | 633 | - | MD **23.91 lower** (31.37 lower to 16.46 lower) | ⨁⨁◯◯ Low |  |
| **ASA Classification (greater than or equal to 3)** | | | | | | | | | | | | |
| 3 | observational studies | not serious | not serious | not serious | not serious | none | 37/141 (26.2%) | 179/514 (34.8%) | **OR 0.80** (0.42 to 1.52) | **49 fewer per 1,000** (from 165 fewer to 100 more) | ⨁⨁◯◯ Low |  |
| **Neutrophil Lymphocyte Ratio** | | | | | | | | | | | | |
| 1 | observational studies | not serious | serious^c^ | not serious | not serious | none | 38 | 133 | - | MD **0.78 lower** (1.36 lower to 0.19 lower) | ⨁◯◯◯ Very low |  |
|  | | | | | | | | | | | | |
| **Alcohol Use** | | | | | | | | | | | | |
| 2 | observational studies | not serious | serious^c^ | not serious | serious^b^ |  | 2/103 (1.9%) | 28/424 (6.6%) | **OR 0.39** (0.04 to 3.61) | **39 fewer per 1,000** (from 63 fewer to 137 more) | - |  |
| **Biliary Stenting** | | | | | | | | | | | | |
| 3 | observational studies | not serious | very serious^a^ | not serious | not serious | none | 47/122 (38.5%) | 294/569 (51.7%) | **OR 0.62** (0.24 to 1.61) | **118 fewer per 1,000** (from 313 fewer to 116 more) | ⨁◯◯◯ Very low |  |

**CI:** confidence interval; **MD:** mean difference; **OR:** odds ratio

#### Explanations

a. Substantial heterogeneity (I^2 > 75%)

b. Wide confidence interval

c. Moderate heterogeneity (I^2 > 50%)

**Tumour Characteristics**

| **Certainty assessment** | | | | | | | **№ of patients** | | **Effect** | | **Certainty** | **Importance** |
| --- | --- | --- | --- | --- | --- | --- | --- | --- | --- | --- | --- | --- |
| **№ of studies** | **Study design** | **Risk of bias** | **Inconsistency** | **Indirectness** | **Imprecision** | **Other considerations** | **Long-Term Survivors** | **Non-Long-Term Survivors** | **Relative (95% CI)** | **Absolute (95% CI)** |  |  |
| **Tumour Location (Pancreatic Head vs. Other Locations)** | | | | | | | | | | | | |
| 12 | observational studies | not serious | not serious | not serious | not serious | none | 1154/1516 (76.1%) | 13918/18115 (76.8%) | **OR 0.96** (0.84 to 1.09) | **7 fewer per 1,000** (from 32 fewer to 15 more) | ⨁⨁◯◯ Low |  |
| **Tumour Grade (≥Grade II vs. Grade I)** | | | | | | | | | | | | |
| 9 | observational studies | not serious | not serious | not serious | not serious | strong association | 1146/1417 (80.9%) | 15151/16830 (90.0%) | **OR 0.40** (0.30 to 0.54) | **117 fewer per 1,000** (from 170 fewer to 71 fewer) | ⨁⨁⨁◯ Moderate |  |
| **Tumour Grade (≥Grade III vs. <Grade III)** | | | | | | | | | | | | |
| 17 | observational studies | not serious | serious^a^ | not serious | not serious | strong association | 432/1720 (25.1%) | 6975/18071 (38.6%) | **OR 0.57** (0.43 to 0.74) | **122 fewer per 1,000** (from 173 fewer to 68 fewer) | ⨁⨁◯◯ Low |  |
| **Tumour Size (cm)** | | | | | | | | | | | | |
| 6 | observational studies | not serious | very serious^b^ | not serious | serious^c^ | none | 297 | 1269 | - | MD **1.45 lower** (3.19 lower to 0.3 higher) | ⨁◯◯◯ Very low |  |
| **AJCC/UICC Stage (≥Stage IIB vs. <Stage IIB)** | | | | | | | | | | | | |
| 14 | observational studies | not serious | not serious | not serious | not serious | very strong association | 377/871 (43.3%) | 8372/12412 (67.5%) | **OR 0.36** (0.31 to 0.41) | **247 fewer per 1,000** (from 283 fewer to 215 fewer) | ⨁⨁⨁⨁ High |  |
| **AJCC/UICC Stage (≥Stage III vs. <Stage III)** | | | | | | | | | | | | |
| 14 | observational studies | not serious | not serious | not serious | not serious | very strong association | 38/875 (4.3%) | 1769/12513 (14.1%) | **OR 0.29** (0.20 to 0.41) | **96 fewer per 1,000** (from 109 fewer to 78 fewer) | ⨁⨁⨁⨁ High |  |
| **Lymphatic Invasion** | | | | | | | | | | | | |
| 6 | observational studies | not serious | not serious | not serious | not serious | strong association | 107/280 (38.2%) | 736/1290 (57.1%) | **OR 0.44** (0.32 to 0.60) | **202 fewer per 1,000** (from 272 fewer to 127 fewer) | ⨁⨁⨁◯ Moderate |  |
| **Perineural Invasion** | | | | | | | | | | | | |
| 10 | observational studies | not serious | serious^a^ | not serious | not serious | strong association | 242/480 (50.4%) | 1357/2087 (65.0%) | **OR 0.46** (0.29 to 0.72) | **195 fewer per 1,000** (from 300 fewer to 88 fewer) | ⨁⨁◯◯ Low |  |
| **Vascular Invasion** | | | | | | | | | | | | |
| 10 | observational studies | not serious | not serious | not serious | not serious | strong association | 150/464 (32.3%) | 925/2062 (44.9%) | **OR 0.52** (0.41 to 0.66) | **151 fewer per 1,000** (from 198 fewer to 99 fewer) | ⨁⨁⨁◯ Moderate |  |
| **Lymph Node Metastasis** | | | | | | | | | | | | |
| 17 | observational studies | not serious | not serious | not serious | not serious | very strong association | 431/1031 (41.8%) | 7825/12179 (64.2%) | **OR 0.40** (0.35 to 0.46) | **224 fewer per 1,000** (from 256 fewer to 190 fewer) | ⨁⨁⨁⨁ High |  |
| **Pathologic T-stage (≥T3 vs. <T3)** | | | | | | | | | | | | |
| 12 | observational studies | not serious | not serious | not serious | not serious | strong association | 605/932 (64.9%) | 9377/12851 (73.0%) | **OR 0.40** (0.30 to 0.53) | **211 fewer per 1,000** (from 282 fewer to 141 fewer) | ⨁⨁⨁◯ Moderate |  |
| **Pathologic M-Stage (Distant Metastases)** | | | | | | | | | | | | |
| 4 | observational studies | not serious | not serious | not serious | not serious | very strong association | 4/545 (0.7%) | 598/11084 (5.4%) | **OR 0.16** (0.06 to 0.38) | **45 fewer per 1,000** (from 51 fewer to 33 fewer) | ⨁⨁⨁⨁ High |  |

**CI:** confidence interval; **MD:** mean difference; **OR:** odds ratio

#### Explanations

a. Moderate heterogeneity (I^2 > 50%)

b. Substantial heterogeneity (I^2 > 75%)

c. Wide CI

| **Certainty assessment** | | | | | | | **№ of patients** | | **Effect** | | **Certainty** | **Importance** |
| --- | --- | --- | --- | --- | --- | --- | --- | --- | --- | --- | --- | --- |
| **№ of studies** | **Study design** | **Risk of bias** | **Inconsistency** | **Indirectness** | **Imprecision** | **Other considerations** | **Long-Term Survivors** | **Non-Long-Term Survivors** | **Relative (95% CI)** | **Absolute (95% CI)** |  |  |
| **Operative Time (minutes)** | | | | | | | | | | | | |
| 4 | observational studies | not serious | not serious | not serious | not serious | strong association | 160 | 639 | - | MD **33.88 lower** (59.6 lower to 8.16 lower) | ⨁⨁⨁◯ Moderate |  |
| **Intraoperative Radiotherapy** | | | | | | | | | | | | |
| 2 | observational studies | not serious | not serious | not serious | not serious | none | 27/58 (46.6%) | 146/358 (40.8%) | **OR 1.20** (0.65 to 2.22) | **45 more per 1,000** (from 99 fewer to 197 more) | ⨁⨁◯◯ Low |  |
| **Perioperative Blood Transfusion** | | | | | | | | | | | | |
| 3 | observational studies | not serious | not serious | not serious | not serious | strong association | 49/122 (40.2%) | 300/526 (57.0%) | **OR 0.50** (0.33 to 0.77) | **171 fewer per 1,000** (from 266 fewer to 65 fewer) | ⨁⨁⨁◯ Moderate |  |
| **Operative Blood Loss (mL)** | | | | | | | | | | | | |
| 4 | observational studies | not serious | serious^a^ | not serious | not serious | strong association | 229 | 735 | - | MD **545.95 lower** (804.52 lower to 287.39 lower) | ⨁⨁◯◯ Low |  |
| **Major Vessel Resection** | | | | | | | | | | | | |
| 7 | observational studies | not serious | not serious | not serious | not serious | none | 66/347 (19.0%) | 359/1301 (27.6%) | **OR 0.62** (0.41 to 0.93) | **85 fewer per 1,000** (from 141 fewer to 14 fewer) | ⨁⨁◯◯ Low |  |
| **Major Vessel Resection - PV Only** | | | | | | | | | | | | |
| 2 | observational studies | not serious | not serious | not serious | not serious | none | 15/56 (26.8%) | 62/219 (28.3%) | **OR 0.79** (0.36 to 1.72) | **45 fewer per 1,000** (from 159 fewer to 121 more) | ⨁⨁◯◯ Low |  |
| **Major Vessel Resection - PV and/or SMV** | | | | | | | | | | | | |
| 2 | observational studies | not serious | very serious^b^ | not serious | not serious | none | 12/101 (11.9%) | 163/591 (27.6%) | **OR 0.51** (0.11 to 2.38) | **113 fewer per 1,000** (from 236 fewer to 200 more) | ⨁◯◯◯ Very low |  |
| **Major Vessel Resection - Any Vascular Resection** | | | | | | | | | | | | |
| 3 | observational studies | not serious | not serious | not serious | not serious | none | 39/190 (20.5%) | 134/491 (27.3%) | **OR 0.71** (0.47 to 1.07) | **62 fewer per 1,000** (from 123 fewer to 14 more) | ⨁⨁◯◯ Low |  |
| **Morbidity (Postoperative Complications)** | | | | | | | | | | | | |
| 3 | observational studies | not serious | not serious | not serious | not serious | none | 22/120 (18.3%) | 69/351 (19.7%) | **OR 0.81** (0.47 to 1.39) | **31 fewer per 1,000** (from 93 fewer to 57 more) | ⨁⨁◯◯ Low |  |
| **Adjuvant Therapy (Chemotherapy only)** | | | | | | | | | | | | |
| 9 | observational studies | not serious | not serious | not serious | not serious | none | 175/318 (55.0%) | 538/1322 (40.7%) | **OR 1.68** (1.03 to 2.74) | **129 more per 1,000** (from 7 fewer to 246 more) | ⨁⨁◯◯ Low |  |
| **Positive Resection Margins** | | | | | | | | | | | | |
| 19 | observational studies | not serious | not serious | not serious | not serious | very strong association | 172/1198 (14.4%) | 3665/13014 (28.2%) | **OR 0.42** (0.35 to 0.50) | **140 fewer per 1,000** (from 161 fewer to 118 fewer) | ⨁⨁⨁⨁ High |  |
| **Hospital Stay (Days)** | | | | | | | | | | | | |
| 3 | observational studies | not serious | serious^a^ | not serious | not serious | none | 141 | 519 | - | MD **0.78 lower** (3.17 lower to 1.61 higher) | ⨁◯◯◯ Very low |  |
| **Adjuvant Therapy (Chemotherapy and/or Radiotherapy)** | | | | | | | | | | | | |
| 13 | observational studies | not serious | not serious | not serious | not serious | none | 528/918 (57.5%) | 5230/12656 (41.3%) | **OR 1.75** (1.29 to 2.38) | **139 more per 1,000** (from 63 more to 213 more) | ⨁⨁◯◯ Low |  |
| **Adjuvant Therapy (Any adjuvant therapy including gemcitabine or nab-paclitaxel)** | | | | | | | | | | | | |
| 6 | observational studies | not serious | serious^a^ | not serious | serious^c^ | none | 124/190 (65.3%) | 371/838 (44.3%) | **OR 2.28** (0.97 to 5.35) | **202 more per 1,000** (from 8 fewer to 367 more) | ⨁◯◯◯ Very low |  |
| **Neoadjuvant Therapy** | | | | | | | | | | | | |
| 4 | observational studies | not serious | very serious^b^ | not serious | not serious | none | 99/198 (50.0%) | 248/597 (41.5%) | **OR 1.48** (0.56 to 3.95) | **97 more per 1,000** (from 131 fewer to 322 more) | ⨁◯◯◯ Very low |  |
| **Neoadjuvant Therapy - Resectable and Borderline Resectable Tumors** | | | | | | | | | | | | |
| 3 | observational studies | not serious | not serious | not serious | not serious | none | 87/160 (54.4%) | 237/464 (51.1%) | **OR 0.99** (0.54 to 1.84) | **3 fewer per 1,000** (from 150 fewer to 147 more) | ⨁⨁◯◯ Low |  |

**CI:** confidence interval; **MD:** mean difference; **OR:** odds ratio

#### Explanations

a. Moderate heterogeneity (I^2 > 50%)

b. Substantial heterogeneity (I^2 > 75%)

c. Wide confidence interval
